# Endothelial cell sphingosine 1-phosphate receptor 1 restrains VE-cadherin cleavage and attenuates experimental inflammatory arthritis

**DOI:** 10.1172/jci.insight.171467

**Published:** 2024-06-10

**Authors:** Nathalie Burg, Ryan Malpass, Linda Alex, Miles Tran, Eric Englebrecht, Andrew Kuo, Tania Pannelini, Margaret Minett, Kalana Athukorala, Tilla Worgall, Heather J. Faust, Susan Goodman, Bella Mehta, Michael Brenner, Dietmar Vestweber, Kevin Wei, Carl Blobel, Timothy Hla, Jane E. Salmon

**Affiliations:** 1 Hospital for Special Surgery, New York, New York, USA.; 2 Division of Rheumatology, Inflammation, and Immunity, Brigham and Women’s Hospital and Harvard Medical School, Boston, Massachusetts, USA.; 3 School of Medicine, University of Louisville, Louisville, Kentucky, USA.; 4 Vascular Biology Program, Boston Children’s Hospital and Department of Surgery, Harvard Medical School, Boston, Massachusetts, USA.; 5 University of Michigan School of Medicine, Ann Arbor, Michigan, USA.; 6 Department of Pathology and Cell Biology, Columbia University, New York, New York, USA.; 7 Max Planck Institute, Munster, Germany.

**Keywords:** Inflammation, Vascular biology, Arthritis, Endothelial cells

## Abstract

In rheumatoid arthritis, inflammatory mediators extravasate from blood into joints via gaps between endothelial cells (ECs), but the contribution of ECs is not known. Sphingosine 1-phosphate receptor 1 (S1PR1), widely expressed on ECs, maintains the vascular barrier. Here, we assessed the contribution of vascular integrity and EC S1PR1 signaling to joint damage in mice exposed to serum-induced arthritis (SIA). EC-specific deletion of S1PR1 or pharmacological blockade of S1PR1 promoted vascular leak and amplified SIA, whereas overexpression of EC S1PR1 or treatment with an S1PR1 agonist delayed SIA. Blockade of EC S1PR1 induced membrane metalloproteinase-dependent cleavage of vascular endothelial cadherin (VE-cadherin), a principal adhesion molecule that maintains EC junctional integrity. We identified a disintegrin and a metalloproteinase domain 10 (ADAM10) as the principal VE-cadherin “sheddase.” Mice expressing a stabilized VE-cadherin construct had decreased extravascular VE-cadherin and vascular leakage in response to S1PR1 blockade, and they were protected from SIA. Importantly, patients with active rheumatoid arthritis had decreased circulating S1P and microvascular expression of S1PR1, suggesting a dysregulated S1P/S1PR1 axis favoring vascular permeability and vulnerability. We present a model in which EC S1PR1 signaling maintains homeostatic vascular barrier function by limiting VE-cadherin shedding mediated by ADAM10 and suggest this signaling axis as a therapeutic target in inflammatory arthritis.

## Abstract 2

Endothelial cell sphingosine 1-phophate receptor 1 restrains VE-cadherin cleavage and attenuates experimental inflammatory arthritis, supporting further evalualation as a potential target for rheumatoid arthritis treatment.

## Graphical abstract

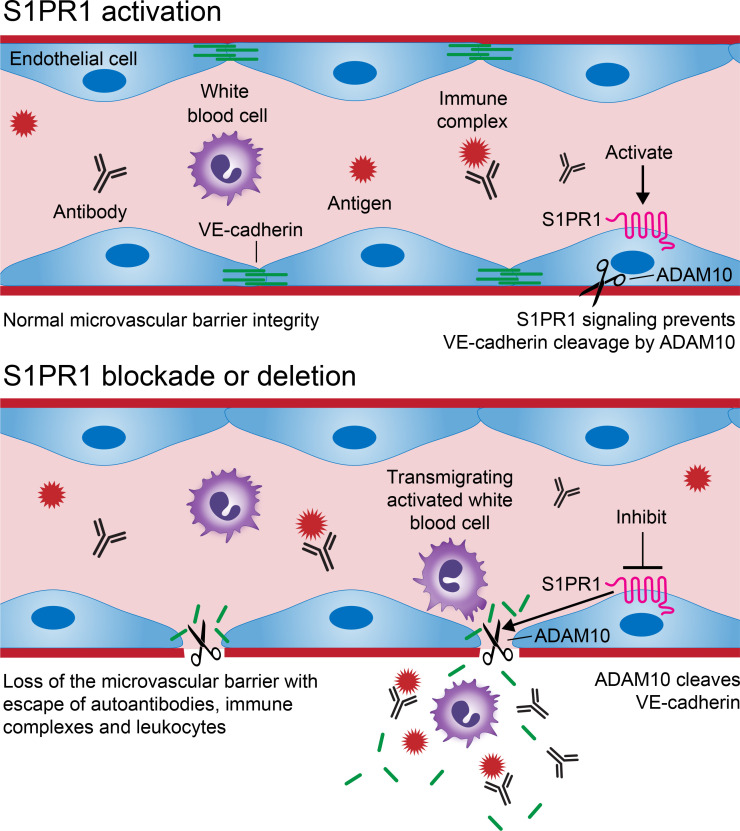

## Introduction

Rheumatoid arthritis (RA) is a debilitating autoimmune disease affecting approximately 0.5%–1% of the world population (1). Despite several FDA-approved drugs for RA, there is an unmet need for new therapeutics, since greater than 30% of patients do not achieve sustained remission (2). Moreover, effective regimens can be complicated by serious infections, and novel agents are needed that can be combined with standard therapies without increasing immunosuppression. Because circulating autoantibodies and immune complexes present in patients with RA (3–5) enter the joint space via intercellular gaps in the synovial microvasculature and recruit and activate effectors of joint damage, we aimed to investigate approaches to target the endothelium in patients suffering from RA and other autoimmune rheumatic diseases.

Endothelial cells (ECs) are the gatekeepers that regulate the extravasation of cells and plasma proteins from the vasculature. Escape of immune complexes (ICs) into the joint space induces inflammation and leukocyte activation (6), and exposure to inflammatory cytokines associated with RA further promotes vascular leakage. ECs modulate inflammation through the maintenance of the vascular barrier and through expression of cytokine-induced pro-adhesive molecules required for leukocyte adhesion and transmigration (i.e., ICAM-1 and VCAM-1) and regulation of extravasation of platelets, platelet microparticles, and other mediators of joint damage (3, 4, 7, 8). In patients with RA, the endothelium is dysfunctional, and markers of EC activation correlate with disease activity (9–13).

Sphingosine 1-phosphate receptor 1 (S1PR1) is a G protein–coupled receptor that is highly expressed on ECs and is a key regulator of homeostatic and vasoprotective functions. Circulating S1P, the major S1PR1 ligand, is produced predominantly by red blood cells and ECs. S1P released into circulation is delivered to S1PR1 on ECs by 2 major chaperones, apolipoprotein M (ApoM), a constituent of high-density lipoprotein (HDL), and albumin. Physiological S1PR1 signaling on ECs (i) maintains barrier integrity and attenuates cytokine-induced vascular leakage (14–17), (ii) inhibits expression of ICAM-1 and VCAM-1 and reduces monocyte adhesion to ECs (18–21), and (iii) inhibits apoptosis (22, 23). ApoM/HDL causes sustained and “biased” signaling in the context of barrier function compared with albumin/S1P (18, 24). Mice lacking ApoM show reduced circulating S1P (25) and increased vulnerability to vascular leak and inflammatory injury (26). To maintain vascular barrier function, S1PR1 signaling acts on the cytoskeleton in a small GTPase Rho/Rac–dependent manner to facilitate translocation of vascular endothelial cadherin (VE-cadherin) and β-catenin to intercellular borders, where they form structures known as adherens junctions (27). VE-cadherins are the principal cell adhesion molecules that maintain EC junctional integrity and thereby regulate permeability and barrier function (28).

In our previous work, we found that signaling of S1PR1 on EC attenuated injury in the reverse passive Arthus reaction, an acute model of IC-mediated injury in skin and lung (17). Increased vascular permeability is a hallmark of the early phase of IC-mediated disease (29–31). We discovered that treatment of cultured ECs with IC-activated neutrophils diminished VE-cadherin expression and decreased EC barrier function. Moreover, pretreatment of ECs with multiple different S1PR1 agonists preserved VE-cadherin expression and barrier integrity (17). These findings suggested that S1PR1 signaling enhanced barrier function, at least in part, by preventing shedding and/or internalization of membrane-bound VE-cadherin. VE-cadherin is known to undergo proteolytic cleavage by metalloproteinases (32–35), notably a disintegrin and a metalloproteinase domain 10 (ADAM10) and ADAM17.

Mouse models of inflammatory arthritis demonstrate that autoreactive immunoglobulins and ICs gain access to synovial tissues due to vascular permeability (31, 36). Mice expressing both the T cell receptor transgene KRN and the MHC class II molecule A(g7) (K/BxN mice) develop severe inflammatory arthritis, and serum from these mice causes a similar arthritis in several mouse strains, due to autoantibodies recognizing glucose-6-phosphate isomerase (37). A rapid vascular leak localized to joints, particularly distal joints, accompanies and promotes subsequent joint damage in the K/BxN serum-induced arthritis (SIA) model that mimics clinical and immunological features of the effector phase of human inflammatory arthritis (29, 38). Similarly, in antigen-induced arthritis, IC entry into the joint space and phagocytosis by synovial lining macrophages initiates neutrophil recruitment and articular inflammation (39).

In the current work, we directly assessed the contribution of vascular integrity and EC S1PR1 signaling to joint damage in SIA. Our results show that S1PR1 attenuates arthritis by increasing microvascular barrier function, and they reveal what we believe is a heretofore-undescribed mechanism for this effect of S1PR1: restraint of a metalloproteinase that cleaves VE-cadherin. We identify ADAM10 as the principal VE-cadherin “sheddase” and show that EC S1PR1 signaling maintains homeostatic barrier function by limiting ADAM10 activity. We also demonstrate that patients with RA show dysregulation of the S1P/S1PR1 axis in ECs in favor of vascular permeability, supporting further studies to specifically target this pathway to treat inflammatory arthritis.

## Results

### Increased vascular permeability persists through the development of SIA and is associated with severity of arthritis.

**Figure 1:**
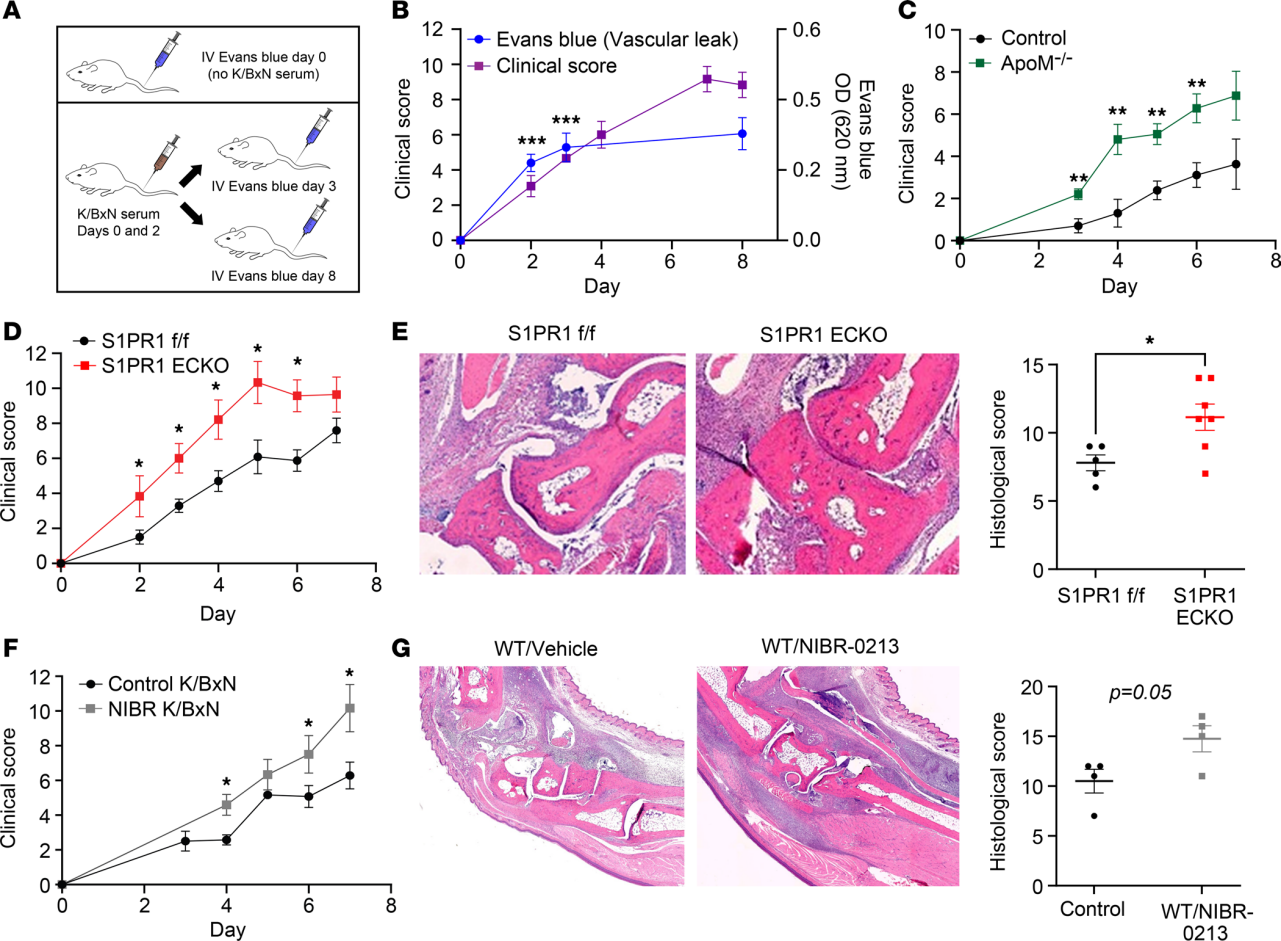
Vascular permeability in SIA is associated with clinical score, and genetic or pharmacological blockade of S1PR1 signaling on ECs worsens SIA. (**A**) Scheme for experiment shown in (**B**): Clinical scores and vascular leakage, as measured by Evans blue extravasation in paws of SIA-treated mice injected with IV Evans blue (0.5% in PBS) 1 hour prior to sacrifice on days 0, 2, 3, and 8 after K/BxN serum injection; *n* = 5–9 mice/group for Evans blue and *n* = 3 mice/group for clinical score. (**C**) Clinical scores from K/BxN serum–treated *ApoM^–/–^* mice and WT controls; *n* = 9 mice/group. (**D**–**G**) Clinical scores, representative H&E-stained paraffin sections of ankle joints, and quantification of histological scores from K/BxN serum–treated mice. Images were scanned at 5× original magnification. (**D** and **E**) S1PR1-ECKO and control mice; *n* = 7–12 mice/group for clinical score; *n* = 5–7 mice for histological score. (**F** and **G**) NIBR-0213– and vehicle control–treated C57BL/6 mice; *n* = 5–7 mice/group for clinical score, *n* = 4 mice for histological score. Significance was calculated using the unpaired 2-tailed Student’s *t* test. Values are the mean ± SEM. *P < 0.05; **P < 0.01; ***P < 0.001.

Administration of K/BxN serum to initiate arthritis induces joint-specific microvascular leak within minutes (31), allowing the passage of autoantibodies and ICs into articular tissues where they initiate damage. To test the hypothesis that vascular permeability is associated with tissue inflammation, we quantified vascular leakage and clinical score of K/BxN SIA. Mice were injected (IV) with Evans blue, which binds circulating albumin, to assess vascular permeability on days 0, 2, 3, and 8 after initiating SIA (Figure 1A). Evans blue extravasation into synovial tissues occurred early in the development of SIA (baseline vs. day 2, *P* = 0.0002; baseline vs. day 3, *P* = 0.0001). Vascular leakage was evident at days 2, 3, and 8 (Figure 1B), supporting a role for early and continuing vascular escape of inflammatory mediators in promoting joint damage. During the resolution phase of SIA, Evans blue leakage decreased (SIA day 18 vs. day 8, 0.3 ± 0.14 OD units vs. 0.14 ± 0.06, respectively; *n* = 10 paws from 5 mice/group; *P* = 0.004).

### Blockade of S1PR1 signaling on ECs amplifies SIA.

Because S1P signaling preserves EC barrier function, we hypothesized that *Apom^–/–^* mice, which have an approximately 50% reduction in circulating S1P and show increased injury in a carrageenan-induced model of inflammation (25, 26), would be more vulnerable to SIA. Indeed, *Apom^–/–^* mice had greater clinical scores than wild-type (WT) mice (Figure 1C), and microCT evaluation of paws from *Apom^–/–^* mice showed modest but statistically decreased bone volume compared with WT mice on day 7 of SIA, indicative of more severe joint damage (Supplemental Figure 1; supplemental material available online with this article; https://doi.org/10.1172/jci.insight.171467DS1). These findings support a key role for S1P signaling to protect from SIA, but they do not exclude protective effects of ApoM that are independent of S1P delivery.

To directly test the hypothesis that S1PR1 signaling on ECs protects against the development of SIA, we subjected mice with an EC-specific deletion of S1PR1 (S1PR1-ECKO mice) to SIA. Western blotting confirmed efficient deletion of S1PR1 in lung tissues (Supplemental Figure 2), and RNA-Seq data of sorted synovial ECs also demonstrated deletion of S1PR1 (Supplemental Figure 3). S1PR1-ECKO mice treated with K/BxN serum showed more severe arthritis measured by clinical score and more inflammation and joint damage assessed histologically compared with tamoxifen-treated littermate controls (S1PR1^fl/fl^) (Figure 1, D and E).

As an alternative strategy to prevent S1PR1 signaling, we induced acute blockade of S1PR1 with NIBR-0213, an S1PR1-specific antagonist shown to induce vascular leakage (40, 41). To determine whether acute S1PR1 blockade exacerbated SIA, mice received NIBR-0213 (30 mg/kg/d) for the first 3 days of SIA. Treatment with NIBR-0213 increased the clinical and histological manifestations of SIA (Figure 1, F and G). The effects of pharmacologic blockade of S1PR1 with NIBR-0213 were comparable to those in S1PR1-ECKO mice (Figure 1, D and G).

### S1PR1 affects the EC transcriptome during inflammatory injury.

EC S1PR1 signaling has been shown to decrease NF-κB signaling as well as ICAM-1 and VCAM-1 expression (18, 42). Therefore, we hypothesized that deficiency in S1PR1 would alter EC gene expression and amplify the pro-inflammatory endothelial phenotype induced during SIA. To identify genes and pathways affected by S1PR1, synovial ECs were isolated by flow sorting from S1PR1-ECKO mice and controls on day 7 of SIA, and transcriptomes were profiled by bulk RNA-Seq. ECs from S1PR1-ECKO mice showed increased proinflammatory transcriptomes, with higher C3, C4b, TNF, CSF-1, and VCAM-1 (Supplemental Figure 3). They also showed upregulated pathways of cytokine signaling in immune system, focal adhesion, and glycosaminoglycan metabolism (Supplemental Figure 4), suggesting that ECs are active participants contributing to inflammatory injury during SIA.

### Amplification of EC S1PR1 signaling mitigates SIA.

**Figure 2:**
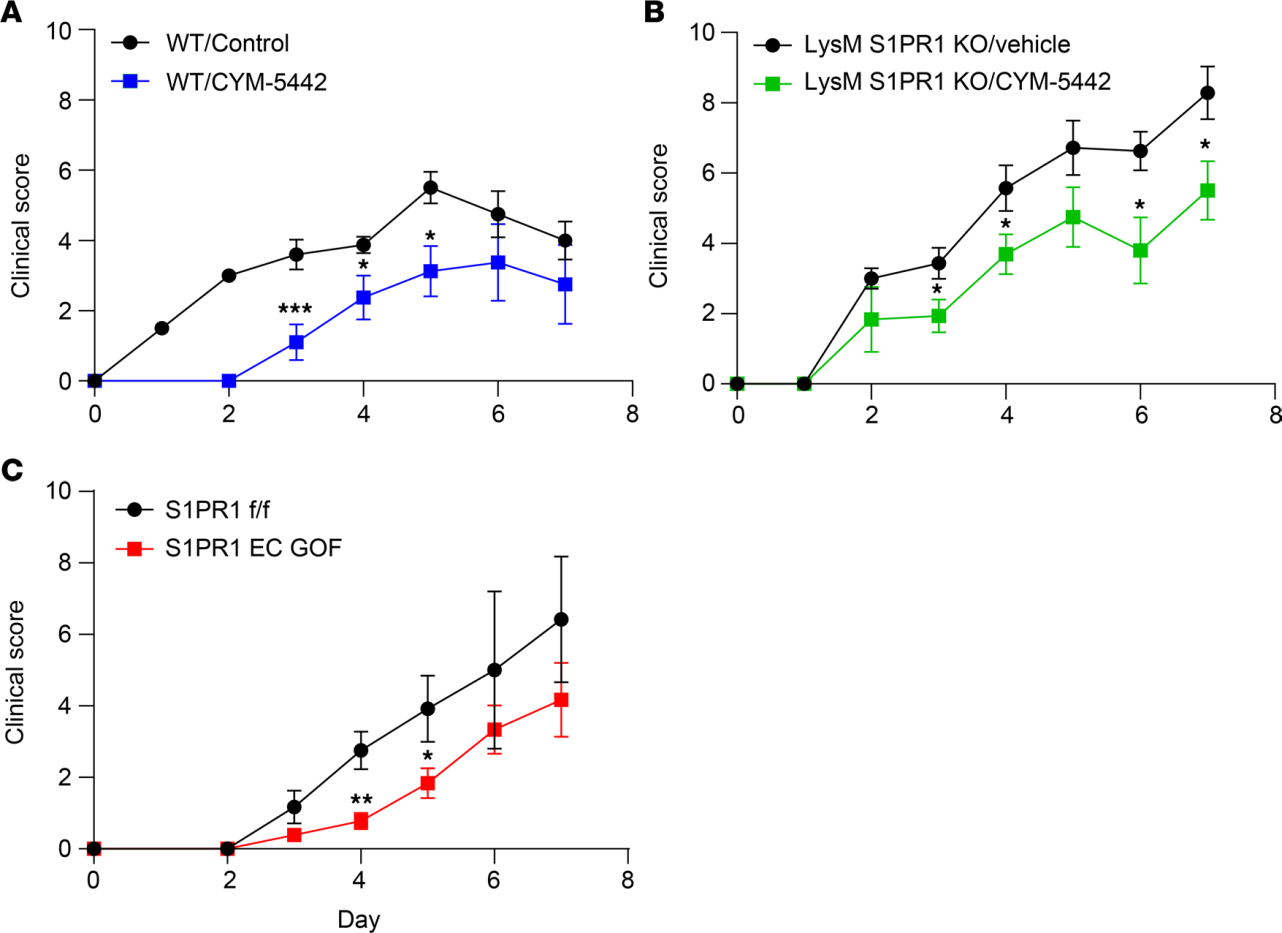
Pharmacologic and genetic enhancement of EC barrier function delays onset and attenuates severity of SIA. (**A**) Clinical scores of WT C57BL/6 mice subjected to SIA, treated with CYM-5442 (0.25 mg/kg IP daily) or vehicle for 7 days; *n* = 5 mice/group. (**B**) Clinical scores of mice with a myeloid-specific KO of S1PR1 (LysM S1PR1 KO) or littermate controls treated with CYM-5442 or vehicle for 7 days; *n* = 7–8 mice/group. (**C**) Clinical scores of mice with tamoxifen-inducible EC gain of function (GOF) of S1PR1 versus tamoxifen-treated controls; *n* = 5–7 mice/group. Significance was calculated using the unpaired 2-tailed Student’s *t* test. Clinical score values are mean ± SEM. *P < 0.05; **P < 0.01; ***P < 0.001.

To determine whether increasing S1P signaling on ECs reduces the progression of SIA, we used pharmacologic and genetic approaches. In prior work, we showed that low-dose CYM-5442, which functions as an S1PR1 agonist, augmented vascular barrier integrity in vitro and attenuated the reverse Arthus reaction, an IC-mediated injury of skin and lung (17). Administration of low-dose CYM-5442 (0.25 mg/kg) on days 0–7 after transfer of K/BxN serum significantly delayed the onset of arthritic injury assessed by clinical scores (Figure 2A).

Although S1PR1 expression is high in ECs, the receptor is also present on myeloid cells, which contribute to the pathogenesis of SIA (38, 43). To exclude the possibility that the protective effect of CYM-5442 in SIA was mediated by increased S1PR1 signaling in myeloid cells, we tested whether CYM-5442 attenuated SIA in mice with a myeloid-specific deletion of S1PR1 (S1PR1 LysM Cre-KO). First, we determined that the clinical scores of SIA were similar in S1PR1 LysM Cre-KO and S1PR1^fl/fl^ littermate controls (data not shown). As in WT mice, CYM-5442 attenuated severity of SIA in S1PR1 LysM Cre-KO mice (Figure 2B), establishing that the protective effects of CYM-5442 do not depend on engagement of S1PR1 on myeloid cells.

As an additional approach to verify that S1PR1 signaling on ECs attenuates SIA, we crossed *S1pr1^fl/stop/fl^* to cadherin 5–CreERT2 heterozygous (Cdh5-CreERT2^+/–^) mice to generate EC S1PR1 gain-of-function (GOF) mice and controls (44). The onset of SIA was also delayed in S1PR1 EC GOF mice (Figure 2C). As shown in other strains exposed to SIA and interventions that alter S1PR1 signaling, clinical scores among different experimental groups converged at later time points. Results in S1PR1-ECKO and GOF mice (Figures 1 and 2) mirror those with pharmacologic agents targeted at S1PR1, supporting a role of S1PR1, rather than other S1PRs, and they argue against attributing modulation of SIA to off-target effects. Taken together, these data indicate that signaling by S1PR1 on ECs delays inflammatory injury in response to SIA.

### Early in SIA, VE-cadherin is shed from the microvasculature into synovial fluid, and levels are increased in S1PR1-ECKO mice.

**Figure 3:**
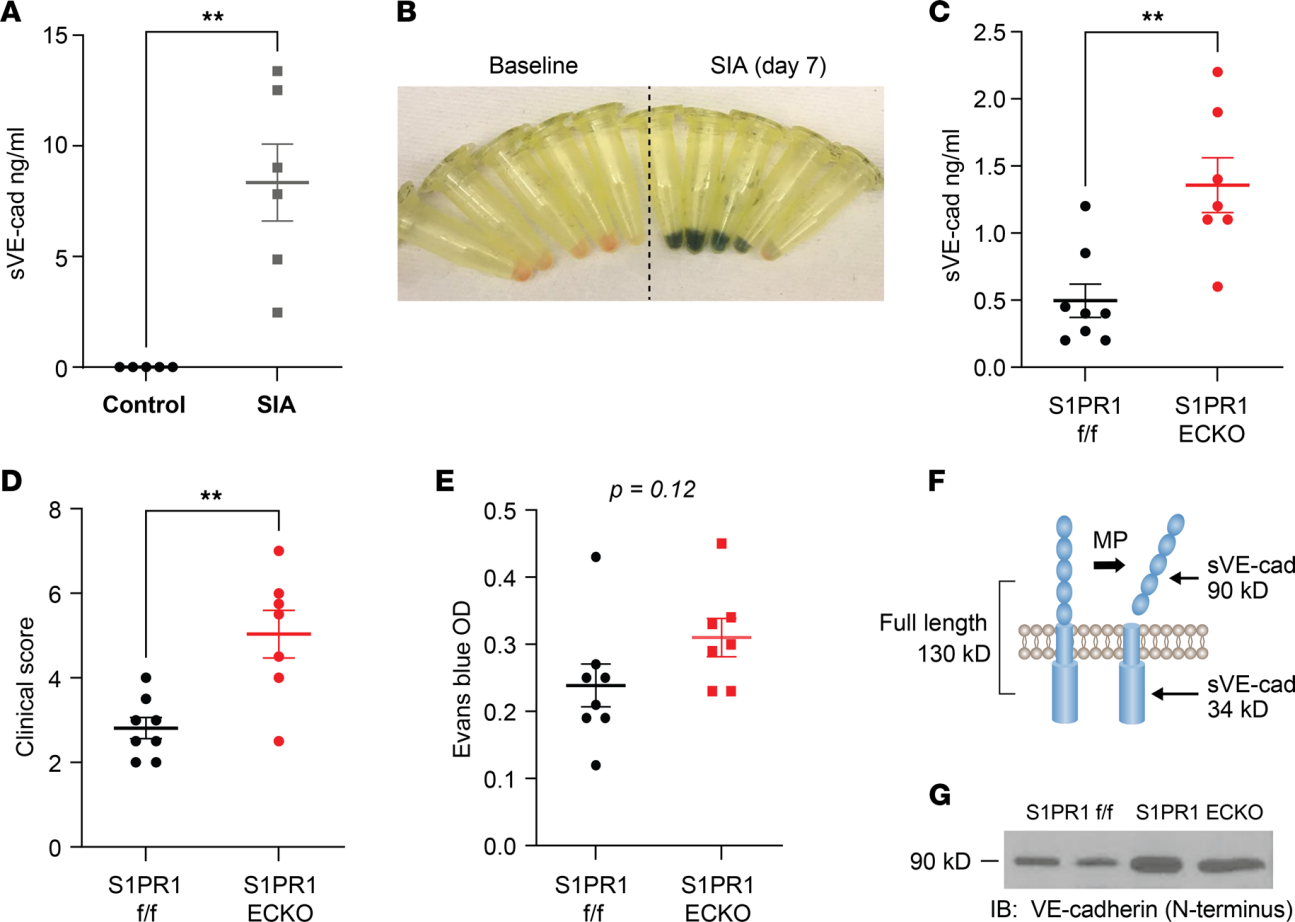
VE-cadherin is shed from ECs and released into synovial fluids during SIA, and levels are increased in S1PR1-ECKO mice. (**A**) Synovial fluid VE-cadherin in SIA-treated mice on day 7 versus untreated controls; *n* = 5–6 mice/group. Each dot corresponds to a sample from an individual mouse. (**B**) Image of synovial lavage fluids from mice injected with Evans blue IV from panel **A**; *n* = 5 mice/group. (**C**) Synovial VE-cadherin at 2–3 days after SIA in S1PR1-ECKO mice versus controls; *n* = 7–8 mice/group. (**D**) Clinical scores of S1PR1-ECKO mice and littermate controls at days 2–3 after SIA; *n* = 7–8 mice/group. (**E**) EB extravasation in synovial tissues on days 2–3 after SIA; *n* = 7–8 mice/group. (**F**) Model of metalloproteinase-mediated VE-cadherin cleavage resulting in the release of a 90 kDa fragment. MP, metalloproteinase. (**G**) Representative Western blot of synovial fluids isolated from S1PR1-ECKO mice and controls probed with an antibody targeting the N-terminal portion of VE-cadherin. Significance was calculated using the unpaired Student’s *t* test. Bars represent means ± SEM. **P < 0.01 or as indicated.

VE-cadherin is a key mediator of adherens junctions and vascular permeability (45, 46). We have shown previously that pretreatment of HUVECs with S1PR1 agonists attenuates IC-induced loss of barrier integrity and the associated decrease in surface VE-cadherin in ECs (17). These findings suggested that S1PR1 signaling limits internalization and/or shedding of VE-cadherin. Because soluble VE-cadherin has been shown to correlate with disease activity in RA (33, 47), we considered the possibility that VE-cadherin is shed by a metalloproteinase(s) during SIA and that S1PR1-ECKO mice have increased arthritis, at least in part, because of enhanced shedding of VE-cadherin in the synovial microvasculature. First, we determined whether soluble VE-cadherin was present in synovial fluids after SIA in WT mice on day 7, corresponding to peak clinical scores. We found elevated levels compared with those in joint lavage from untreated mice (Figure 3A), which was accompanied by increased Evans blue extravasation (Figure 3B).

To determine whether S1PR1 signaling on ECs limits VE-cadherin shedding, we compared levels of soluble VE-cadherin in synovial tissues from S1PR1-ECKO mice and S1PR1^fl/fl^ littermate control mice treated with K/BxN serum. Early in the development of SIA (days 2–3), S1PR1-ECKO mice showed 3-fold higher soluble VE-cadherin levels in synovial fluid (Figure 3C). In contrast, plasma levels of soluble VE-cadherin were only 34% higher in S1PR1-ECKO mice compared with controls (Supplemental Figure 5). S1PR1-ECKO mice also had significantly higher clinical scores and a trend toward increased synovial Evans blue extravasation at days 2–3 (Figure 3, D and E). Once joint inflammation peaked at days 7–8 and clinical differences between S1PR1-ECKO mice and WT were smaller (Figure 1D), there were no longer detectable differences in soluble VE-cadherin levels in synovial fluids between S1PR1-ECKO mice and WT (data not shown).

Soluble VE-cadherin is present in full-length (130 kDa) and cleaved (90 kDa) forms (Figure 3F), corresponding to membrane-associated or metalloproteinase-cleaved shed protein, respectively. To distinguish between these forms, we performed Western blots of synovial fluids from SIA mice probed with an antibody specific to the N-terminal portion of VE-cadherin (extracellular region). VE-cadherin in synovial fluid contained only the 90 kDa protein corresponding to cleaved VE-cadherin (Figure 3G and Supplemental Figure 6). Importantly, synovial fluid from SIA-treated S1PR1-ECKO mice demonstrated more of the 90 kDa shed form of VE-cadherin than littermate controls (Figure 3G).

### EC S1PR1 signaling restrains an EC metalloproteinase that cleaves VE-cadherin to maintain homeostatic vascular barrier function.

**Figure 4:**
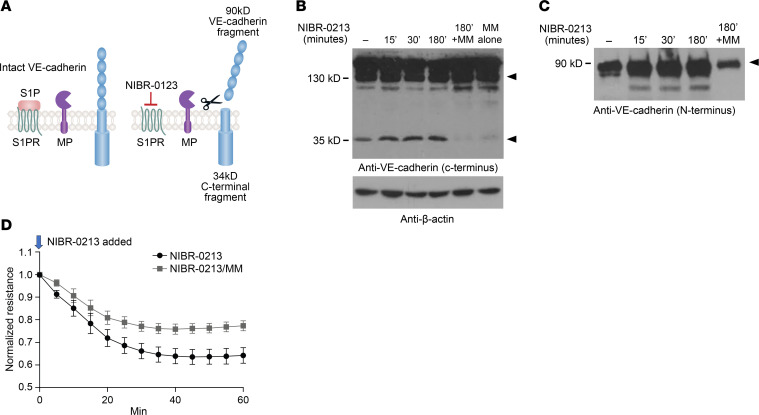
S1PR1 signaling restrains VE-cadherin shedding. (**A**) Proposed model: S1PR1 signaling restrains the metalloproteinase-mediated cleavage of VE-cadherin. (**B**) Western blot of lysates from HUVECs treated with NIBR-0213 (10 μM) or vehicle and probed with a C-terminal–specific anti–VE-cadherin antibody (top panel) or β-actin (bottom panel). Representative image of *n* = 3. MM, marimastat. (**C**) Western blot of supernatants from HUVECs treated with 10 μM NIBR-0213 with or without MM (1 μM) for indicated times; glycosylated proteins were concentrated with concanavalin A beads, and eluates were probed with an N-terminal–specific antibody to VE-cadherin. (**D**) HUVECs treated with NIBR-0213 (0.5 μM) in the presence or absence of MM (1 μM) were subjected to electric cell-substrate impedance sensing (ECIS). Values are the mean ± SEM; *n* = 4 independent experiments. Significance was calculated using the unpaired 2-tailed Student’s *t* test. **P < 0.01.

VE-cadherin can be cleaved from the EC surface by membrane-associated “sheddases,” such as ADAM10 (34) or ADAM17 (35), or by soluble proteases originating from ECs and other cell types, particularly neutrophils and other myeloid cells (48). We hypothesized that S1PR1 signaling restrained metalloproteinase-induced shedding of VE-cadherin. To directly test the possibility proposed in Figure 4A that S1PR1 blockade induces the metalloproteinase-dependent cleavage of VE-cadherin, we treated HUVECs with the S1PR1 antagonist NIBR-0213 (10 μM) for 30–180 minutes and assessed generation of C-terminal (intracellular domain) in cell lysates and N-terminal shed VE-cadherin fragments in supernatants with Western blots. Within 15 minutes of NIBR-0213 treatment, there was an increase in the 35 kDa cell-bound C-terminal fragment (Figure 4B), along with an increase in the 90 kDa shed fragment of VE-cadherin in the supernatants (Figure 4C).

To determine whether an EC-associated metalloproteinase cleaves VE-cadherin (34, 35), we performed experiments in the presence or absence of a nonspecific hydroxamate-type metalloproteinase inhibitor MM. Pretreatment with MM blocked NIBR-0213–induced shedding of VE-cadherin in HUVECs. The cell-bound 35 kDa C-terminal fragment and the soluble N-terminal 90 kDa VE-cadherin fragment released into supernatants decreased (Figure 4, B and C). These data support the notions that S1PR1 signaling on ECs maintains intact cell surface VE-cadherin and that VE-cadherin shedding induced by S1PR1 blockade is dependent upon metalloproteinase(s).

To test whether metalloproteinase-induced shedding alters vascular barrier function, we used electrical cell-substrate impedance sensing (ECIS), which quantifies EC barrier function. Blockade of S1PR1 signaling with NIBR-0213 caused a decline in baseline resistance within 5–10 minutes, consistent with EC barrier disruption, and treatment with MM attenuated the decrease in resistance induced by NIBR-0213 (Figure 4D). We verified these data in ECIS using synovial ECs (Supplemental Figure 7). Addition of MM had no detectable effect on baseline resistance when added 30–60 minutes before NIBR-0213. Our findings provide functional evidence that loss of EC barrier function induced by S1PR1 blockade requires the activity of a metalloproteinase that cleaves VE-cadherin and/or another metalloproteinase substrate and suggest that S1PR1 restrains the metalloproteinase-dependent vascular permeability.

**Figure 5:**
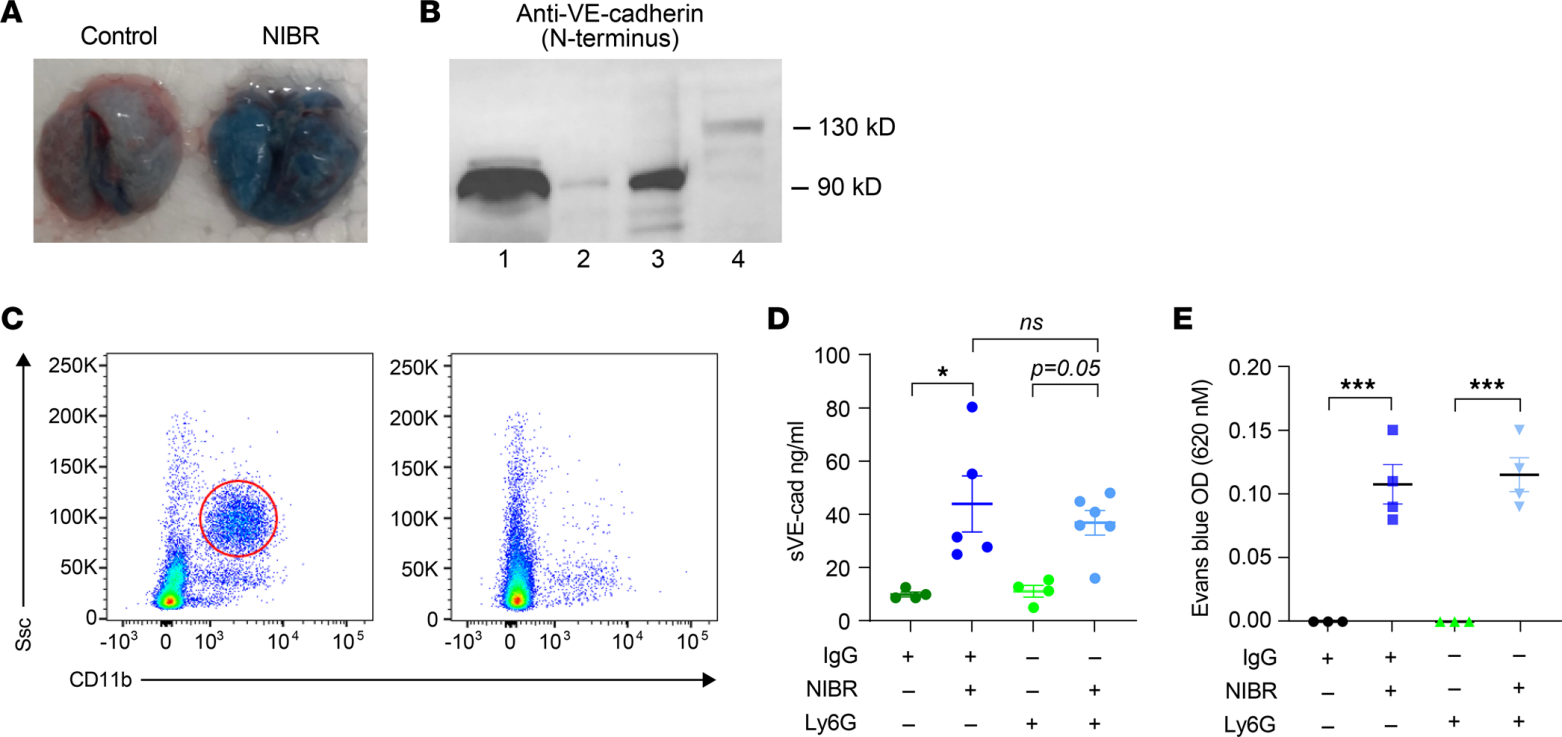
S1PR1 blockade induces VE-cadherin shedding and vascular leak in the lung that does not depend on neutrophils. (**A**) Representative image of perfused lungs from mice injected with Evans blue in the presence of NIBR-0213 or vehicle control. (**B**) Representative Western blot of (lane 1) plasma or (lane 2) BAL fluid from an untreated mouse, (lane 3) BAL fluid from an NIBR-0213–treated mouse, and (lane 4) HUVEC lysate indicating that in both plasma and BAL fluids, VE-cadherin is present as the cleaved 90 kDa fragment. Data represent at least 2 independent experiments. (**C**) Flow cytometry verification that anti-Ly6G antibodies depleted CD11b^+^ neutrophils. Left panel: isotype control–treated mice. Right panel: anti-Ly6G–treated mice. (**D**) Soluble VE-cadherin in BAL fluid from mice treated with isotype control antibody or anti-Ly6G 1 day prior to treatment with NIBR-0213; *n* = 4–5 mice/group. (**E**) Evans blue in BAL fluids of mice treated with isotype control antibody or anti-Ly6G 1 day prior to treatment with NIBR-0213. Each point represents an individual mouse. Statistical test was 1-way ANOVA with Tukey’s post hoc test; *P < 0.05; ***P < 0.001; or as indicated.

To verify the in vitro findings in mice with pharmacologic blockade of S1PR1, we treated WT mice with NIBR-0213 (30 mg/kg) and assessed vascular integrity in the lung by measuring Evans blue extravasation and soluble VE-cadherin in bronchoalveolar lavage (BAL). Three hours after IP administration of NIBR-0213, there was an increase in Evans blue extravasation in the lungs and in BAL fluid (Figure 5A). We verified by Western blot that the soluble VE-cadherin present in BAL was the 90 kDa cleaved form (Figure 5B). Our in vitro findings indicated that NIBR-0213 acted directly on ECs, but systemic S1PR1 blockade may affect many cells. Neutrophil-derived mediators have been implicated in VE-cadherin shedding (48). To determine whether neutrophils and the proteolytic enzymes they release contributed to NIBR-0213–induced VE-cadherin shedding by ECs and vascular leakage, we treated FVN/B mice, which have been shown to have efficient neutrophil depletion after treatment with anti-Ly6G antibodies (49), 1 day before administration of NIBR-0213. Depletion of neutrophils was verified by flow cytometry (Figure 5C). The increase in soluble VE-cadherin in BAL in neutrophil-depleted mice was similar to that in mice treated with isotype control antibody (Figure 5D), indicating shedding of VE-cadherin induced by S1PR1 blockade does not require the presence of neutrophils. We verified the myeloid depletion studies in C57BL/6 mice, the strain used in our SIA experiments, which are known to resist anti-Ly6G depletion (50). We treated mice with anti-mouse granulocyte receptor-1 (anti-GR1) antibodies 1 day before challenge with NIBR-0213. Evans blue extravasation and soluble VE-cadherin increased in BAL in neutrophil-depleted mice, albeit somewhat less than in mice that did not receive anti-GR1 (Supplemental Figure 8). This may be the case because GR1 is more broadly expressed than Ly6G and has been shown to be present on some ECs (51).

### VE-cadherin-α-catenin–knockin mice show reduced vascular leakage in response to pharmacological inhibition of S1PR1 and attenuated SIA.

**Figure 6:**
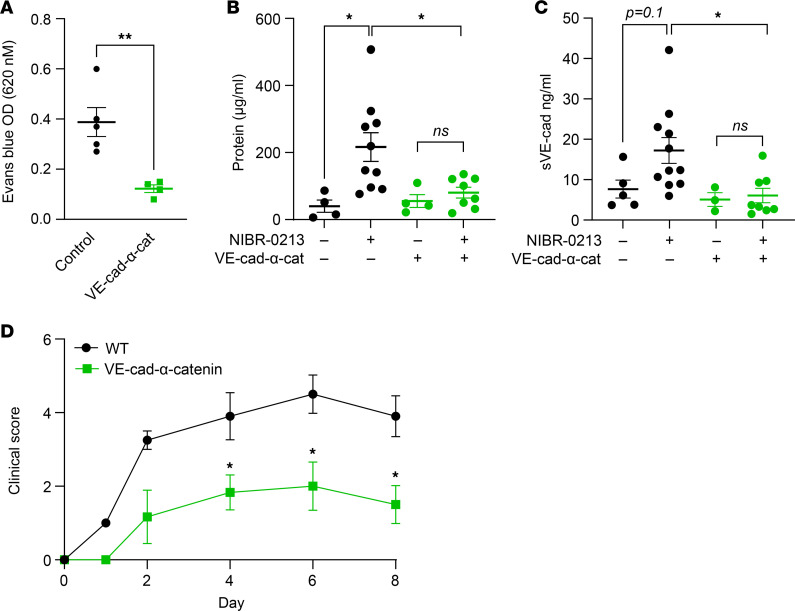
Mice with a stabilized VE-cadherin construct resist vascular leakage and VE-cadherin shedding and have attenuated SIA. (**A**) Evans blue extravasation in skin 30 minutes after subcutaneous histamine injection in VE-cad-α-cat mice and controls; *n* = 4–5 mice/group. (**B**) BAL protein, *n* = 4–10 mice/group; and (**C**) BAL soluble VE-cadherin, *n* = 3–11 mice/group from mice treated with NIBR-0213 or vehicle control. (**D**) Clinical scores of VE-cad-α-cat mice and controls subjected to SIA; *n* = 8–9 mice/group. *P < 0.05; **P < 0.01; ns, not significant; or as indicated. Significance was calculated using the unpaired 2-tailed Student’s *t* test (**A** and **D**) or 1-way ANOVA and Tukey’s post hoc test (**B** and **C**).

Given the key role of VE-cadherin in the maintenance of microvascular barrier function (28, 52), we asked whether the vascular leakage induced by blocking S1PR1 was mediated, at least in part, by cleavage of VE-cadherin. To answer this question, we used a gene-targeted mouse in which VE-cadherin is replaced by a construct of VE-cadherin fused to α-catenin (VE-cad-α-cat mice). This mutation leads to highly stabilized EC junctions that resist vascular leak in response to histamine and VEGF (53). We verified that homozygous VE-cad-α-cat mice were protected from histamine-induced vascular barrier disruption in the skin compared with heterozygous controls (Figure 6A) and discovered that they resisted NIBR-0213–induced vascular permeability. While mice heterozygous for the VE-cad-α-cat fusion construct showed extravasation of protein into the BAL fluid after treatment with NIBR-0213, homozygous VE-cad-α-cat mice were protected (Figure 6B). Notably, homozygous VE-cad-α-cat mice also did not show a significant increase of soluble VE-cadherin in BAL after NIBR-0213 treatment, whereas heterozygous controls had significantly elevated levels of soluble VE-cadherin in BAL (Figure 6C), though they had similar basal plasma levels of VE-cadherin (Supplemental Figure 9). An alternative explanation for the decrease in soluble VE-cadherin in BAL of homozygous VE-cad-α-cat mice is that the VE-cad-α-cat construct leads to tightening of homotypic interactions of VE-cadherin, such that the extracellular cleavage site targeted by a metalloproteinase is sterically hindered so that VE-cadherin is not shed.

If the VE-cad-α-cat fusion construct caused a more impervious EC barrier and prevented shedding of VE-cadherin and vascular leakage induced by S1PR1 blockade, we expected that VE-cad-α-cat mice would have attenuated SIA. Indeed, homozygous VE-cad-α-cat mice treated with K/BxN serum developed less arthritis than WT controls at the onset and at the peak of inflammation (Figure 6D). Taken together, our findings link S1PR1 blockade leading to cleavage of membrane VE-cadherin with loss of vascular barrier integrity and severity of IC-mediated arthritis.

### EC S1PR1 signaling prevents ADAM10 cleavage of VE-cadherin to maintain homeostatic vascular barrier function.

We next performed a series of experiments to explore the mechanism by which EC S1PR1 limits shedding of VE-cadherin and maintains the vascular barrier. VE-cadherin is a known substrate of the metalloproteinases ADAM10 (32, 34, 54) and ADAM17 (35). MM, a metalloproteinase inhibitor, which blocks both ADAM10 and ADAM17, partially attenuated EC barrier dysfunction induced by S1PR1 blockade (Figure 4D). To assess the contribution of ADAM17, we blocked ADAM17 activity in HUVECs with a neutralizing anti-ADAM17 (150 nM) antibody [D1(A12), IC_50_ 5 nM; ref. 55] and used ECIS to compare the capacity of NIBR-0213 to decrease resistance in cells with and without active ADAM17. Inhibition of ADAM17 activity did not significantly attenuate barrier dysfunction caused by reduction of S1PR1 signaling (Supplemental Figure 10A). As a second approach to inhibit ADAM17 activity, we transfected HUVECs with ADAM17 siRNA (knockdown confirmed by Western blot in Supplemental Figure 10B). Inhibiting expression of ADAM17 on HUVECs did not prevent NIBR-0213–induced shedding of VE-cadherin into cell supernatants (Supplemental Figure 10C). Taken together, these findings indicate that ADAM17 is not required for VE-cadherin shedding and loss of barrier integrity induced by blockade of S1PR1 signaling.

**Figure 7:**
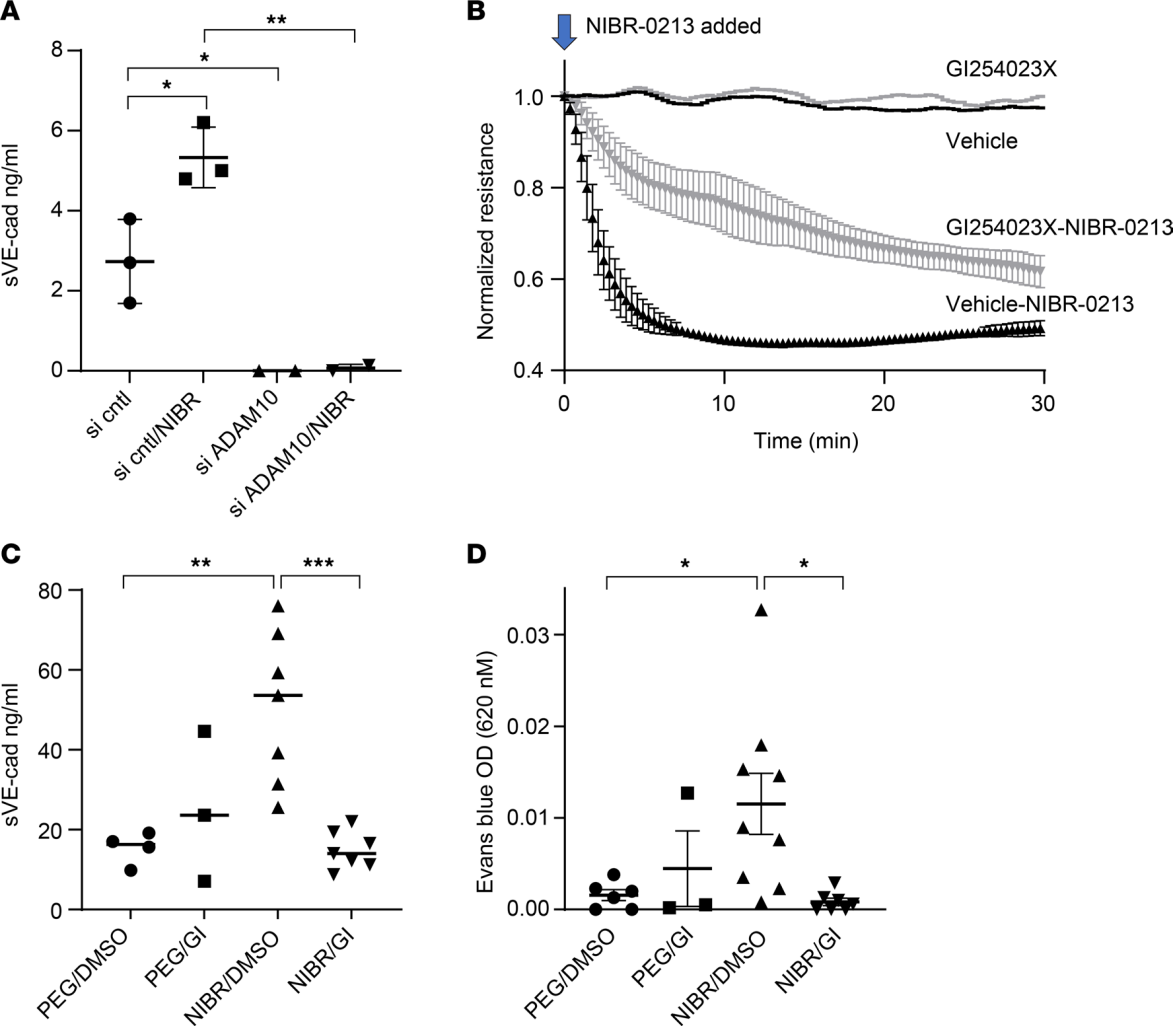
S1PR1 signaling on ECs prevents ADAM10-mediated cleavage of VE-cadherin to maintain barrier function. (**A**) HUVECs were transfected with control or ADAM10 siRNA 2–3 days prior to treatment with NIBR-0213 for 1 hour. Supernatants were collected and soluble VE-cadherin was quantified by ELISA; *n* = 3. (**B**) Synovial endothelial cells were treated with the ADAM10 inhibitor GI254023X (GI) (1 μM) or vehicle (DMSO) for 30–60 minutes prior to treatment with NIBR-0213, and resistance across confluent ECs was measured by ECIS. Values are the mean ± SEM; *n* = 3 independent experiments; ****P ≤ 0.0001. Mice were treated with GI254023X (50 mg/kg IV or DMSO) 30 minutes prior to challenge with NIBR-0213 (10 mg/kg or polyethylene glycol 200, PEG) and BAL fluids were collected. (**C**) Soluble VE-cadherin; *n* = 3–7 mice/group. (**D**) Evans blue; *n* = 3–9 mice/group. Statistical test was 1-way ANOVA with Tukey’s post hoc test; *P < 0.05; **P ≤ 0.01; ***P ≤ 0.001.

We, therefore, focused on ADAM10 as the potential sheddase of VE-cadherin and tested the hypothesis that inhibition of ADAM10 activity would prevent responses of ECs to NIBR-0213. We used genetic and pharmacological approaches to block ADAM10 activity in vitro and in vivo in HUVECs and synovial ECs. In HUVECs transfected with ADAM10 siRNA (knockdown confirmed by Western blot in Supplemental Figure 10B), both basal and NIBR-0213–induced shedding of VE-cadherin were lower than in HUVECs transfected with control siRNA (Figure 7A and Supplemental Figure 10C).

To determine whether inhibition of ADAM10 activity also affects EC barrier function, we performed ECIS assays with synovial ECs pretreated with the ADAM10 inhibitor GI254023X (1 μM). Like HUVECs, synovial ECs responded to NIBR-0213 with a drop in resistance (Figure 7B). Inhibition of ADAM10 activity with GI254023X delayed the decrease in resistance induced by NIBR-0213 (Figure 7B). In the absence of S1PR1 blockade, resistance measured by ECIS was similar in GI254023X- and vehicle-treated ECs (Figure 7B). Overall, these data suggest a relationship between S1PR1 and ADAM10 in the regulation of vascular barrier function.

To determine whether inhibition of ADAM10 prevented shedding of VE-cadherin and vascular leakage induced by S1PR1 blockade in vivo, we assessed responses of lung ECs to S1PR1 blockade with NIBR-0213, similar to experiments in Figures 5 and 6. We treated mice with GI254023X (50 mg/kg IV) or vehicle 30 minutes prior to challenge with NIBR-0213 (10 mg/kg, IP) and measured soluble VE-cadherin and extravasation of Evans blue into BAL fluid. NIBR-0213 induced shedding of VE-cadherin into BAL fluid within 1 hour of its administration, and this was completely abrogated by GI254023X (Figure 7C). Blockade of ADAM10 also attenuated vascular leakage caused by inhibition of S1PR1 signaling as determined by Evans blue extravasation into BAL (Figure 7D). Taken together, our in vitro and in vivo data strongly support a role for S1PR1 signaling in restraining vascular leakage by limiting ADAM10-induced shedding of VE-cadherin.

### Patients with active RA have decreased circulating S1P and decreased expression of synovial EC S1PR1 transcripts compared with patients with osteoarthritis.

**Figure 8:**
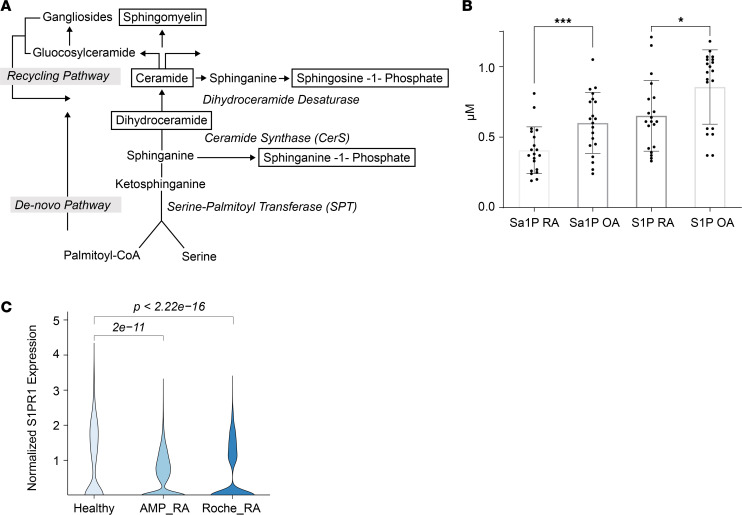
Patients with moderately to severely active RA have lower circulating S1P and decreased S1PR1 expression in synovial capillary ECs. (**A**) Diagram of sphingolipid biosynthesis. (**B**) Sphinganine 1-phosphate (Sa1P) and S1P levels in RA and OA sera. Significance was calculated using the unpaired 2-tailed Student’s t test. Values represent mean ± SEM. (**C**) Violin plots indicating normalized S1PR1 expression in capillaries derived from RA and healthy synovial tissues as determined by single-cell RNA-Seq. *P* values were calculated using pairwise Wilcoxon rank-sum tests. *P < 0.05; ***P ≤ 0.001; or as indicated.

Given the importance of S1PR1 in maintenance of vascular barrier function and our findings in SIA, we sought to determine whether patients with RA have a dysregulated S1P/S1PR1 axis. To address this question, we first compared S1P levels in sera from patients with moderately to severely active RA, as defined as a disease activity score-28 of greater than 3.2, with those from age-, sex-, and race-matched patients with osteoarthritis (OA) (Supplemental Figure 11). Both S1P and sphinganine-1 P, an alternate ligand of S1PR1 (56) (Figure 8A), were significantly lower in patients in RA (Figure 8B).

Next, we compared expression of S1PR1 transcripts in synovial ECs from patients with RA with that in healthy controls using single-cell RNA-Seq from 3 data sets: Accelerating Medicines Partnership Rheumatoid Arthritis/Systemic Lupus Erythematosus (AMP RA/SLE) network (57), the Roche network for RA (58), and Faust et al. (59) for normal synovium. In both data sets of RA synovium, capillary ECs showed significantly decreased S1PR1 transcripts compared with healthy capillary ECs (Figure 8C). Taken together, these data support the concept that patients with RA are more vulnerable to vascular leakage because of loss of protective S1PR1 signaling, secondary to both reduced ligand and receptors, which may contribute to disease severity.

## Discussion

Applying genetic and pharmacologic approaches modulate of S1PR1 signaling, we have established that EC S1PR1 mitigates inflammatory injury in the K/BxN serum transfer model of inflammatory arthritis, which mimics many features of the effector phase of human inflammatory arthritis (29, 38). We found that blockade of EC S1PR1 signaling increased vascular permeability and amplified SIA, whereas augmented EC S1PR1 signaling delayed SIA. We provide what we believe is the first evidence that S1PR1 maintains barrier function, in part, by preventing ADAM10-mediated shedding of VE-cadherin from the EC surface. Impaired microvascular barrier in IC-mediated disease allows entry of inflammatory mediators, soluble factors, and cells into vulnerable tissues. Our findings, taken together with our work in IC-mediated acute injury in skin and lung, highlight a previously unappreciated approach to targeting inflammatory arthritis: augmenting microvascular barrier function.

K/BxN SIA is preceded by a rapid increase in joint-localized permeability of the microvasculature that permits the anti–glucose-6-phosphate isomerase autoantibodies, pathogenic antibodies in K/BxN serum, to exit the circulation and deposit in the joints, where they recruit innate immune cells and activate complement (31, 36). We show that increased vascular permeability is associated with severity of SIA and that S1PR1 signaling limits vascular leakage, VE-cadherin shedding, and joint inflammation, most prominently in the early phase of SIA. Clinical scores, leakage, and VE-cadherin shedding in S1PR1-ECKO mice and controls converged at day 7, suggesting that at the peak of inflammation neutrophils and other inflammatory mediators present in the synovial tissues are principal contributors of vascular leakage. The concept that S1PR1 signaling has the greatest impact on early disease is also supported by our finding that the S1PR1 agonist CYM-5442 attenuates inflammation at early time points more than at later stages of SIA.

That vascular permeability is a driver of inflammatory arthritis is supported by our studies demonstrating that VE-cad-α-cat mice, manifesting stabilized EC contacts and resistance to S1PR1 antagonist–induced vascular leak, have attenuated SIA. Stangenberg et al. reinforce this concept in studies showing that denervation of hind limb nerves prior to SIA prevents inflammation in the paralyzed limb, mimicking the hemiplegia-induced protection from arthritis in patients with RA who have strokes (30). In the mice exposed to SIA, only the neurologically intact side demonstrates inflammation, and the most differentially expressed genes from ECs in enervated versus denervated limbs include representatives of signaling pathways critical for regulating vascular permeability. Importantly, denervated limbs show decreased microvascular leakage after K/BxN serum transfer, and protection from SIA is attributed to changes in EC barrier (30). The notion that a breach in vascular barrier is required for maximal inflammation in autoantibody-induced arthritis is also supported in a model of anti-collagen antibody–induced arthritis in which KO of bradykinin receptors attenuates disease (60).

Our in vivo and in vitro studies underscore the established role of VE-cadherin in maintaining EC barrier integrity. We identify what we believe to be a novel mechanism by which EC S1PR1 mediates its pro-barrier effects — restraining cleavage of VE-cadherin by ADAM10. The mechanisms by which S1PR1 signaling restrains ADAM10-mediated cleavage of VE-cadherin are not yet clear. S1PR1 signaling might restrain the activation of ADAM10, or it could, via its impact on the EC cytoskeleton (27) and VE-cadherin homotypic interactions, limit exposure of VE-cadherin cleavage sites without restraining ADAM10 activity per se. Evidence that S1PR1 signaling protects VE-cadherin against trypsinization supports the latter possibility (44). However, concealment of an ADAM10 cleavage epitope on VE-cadherin by S1PR1 signaling and restraint of ADAM10 activity mediated by S1PR1 are not necessarily mutually exclusive processes. Also, although we showed that ADAM17 is not required for VE-cadherin shedding induced by S1PR1 blockade, we cannot exclude the possibility that ADAM17 contributes.

Our findings support that VE-cadherin shedding affects barrier function, whereas much of the work on VE-cadherin expression has heretofore been focused on its endocytosis and ubiquitination (61–64). Studies to understand the regulation of VE-cadherin cleavage have important clinical implications, as shedding of VE-cadherin has been shown to be a pathogenic mediator in RA (33), sepsis, and other inflammatory states, facilitating both vascular leakage and leukocyte transmigration (32, 34, 65, 66).

We focused on S1PR1 rather than other S1PRs because its expression on ECs is the greatest, and its function is recognized as key to vascular homeostasis (27). S1PR2 and -3 are also expressed on ECs but at lower levels. Recently published work complements our findings relating S1P signaling, ADAM10, VE-cadherin, and vascular barrier. Wu et al. have shown that S1PR3 signaling induces endothelial barrier loss by triggering ADAM10-mediated shedding of VE-cadherin (67). S1PR1 and S1PR3 have opposing roles on vascular barrier function and engage different G proteins, i.e., Gi (S1PR1) versus G12/13 (S1PR3), as recently reviewed (68), and with these new data, they appear to have opposing effects on ADAM10 activity. Whether the opposing roles of S1PR1 and S1PR3 on the Rho/Rac pathway (68) contribute to effects on ADAM10 has yet to be established.

We also found that deletion of S1PR1 in ECs resulted in increased transcription of multiple mediators of inflammatory arthritis. Thus, unrestrained ECs may directly contribute to joint damage by secreting cytokines and other facilitators of tissue injury. This possibility is supported by work showing that S1PR1 inhibits the NF-κB pathway (18, 42). Whether the EC transcriptional changes are linked to increased ADAM10 activity is not yet known. However, production and release of inflammatory cytokines, such as TNF-α and IL-1β, as a consequence of the activity of the metalloproteinase ADAM17 are well described (69), and such mechanisms may drive a positive feedback loop for EC activation. It will be important to determine how S1PR1 blockade impacts the EC transcriptome in the presence or absence of ADAM10 inhibitors.

The mechanisms we have identified as modulators of severity of SIA align with findings in patients with RA. Mice deficient in ApoM, the chaperone on HDL that delivers S1P to S1PR1 and has particularly effective antiinflammatory effects, show amplified arthritis in response to SIA. Similarly, polymorphisms in the *APOM* promoter that result in reduced ApoM levels are associated with an increased risk of RA (70). In SIA, elevations of soluble VE-cadherin in synovial fluids are associated with worse arthritis. In RA patients treated with TNF-α–blocking agents, levels of serum VE-cadherin significantly correlated with C-reactive protein (CRP), a marker of inflammation and disease activity, and were lower in patients whose CRP fell in response to therapy (47).

In contrast with our findings, S1PR1 blockade using NIBR-0213 was shown to be protective in the adjuvant-induced arthritis (AIA) model (40). The protective effects of S1PR1 blockade in the AIA model may be related to decreased lymphocyte trafficking to synovial tissues, which could outweigh pro-permeability effects of NIBR-0213 (71). The K/BxN SIA model does not depend on adaptive immunity, and the clinical and histological worsening we observed in mice treated with NIBR-0213 mice is attributable to amplification of effector mechanisms. Also, in contrast with our studies, S1PR1 blockade attenuates synoviocyte proliferation and production of proinflammatory mediators in vitro (72). Although S1PR1 blockade has selective antiinflammatory effects on non-EC cell types, S1PR1 antagonism is not likely a tenable approach in patients with RA, because treatment with NIBR-0213 during AIA results in pulmonary leakage, inflammation, and fibrosis and patients with RA have a high incidence of clinical and subclinical interstitial lung disease (40).

We report data from single-cell RNA-Seq of human synovium showing that S1PR1 expression on ECs is decreased in RA compared with healthy synovial ECs. We also found lower levels of S1P and sphinganine-1 P, a precursor of S1P and a ligand of S1PR1, in patients with moderately to severely active RA compared with patients with OA. Taken together, our data support the concept that the S1P/S1PR axis is dysregulated in RA in the direction of increased vascular vulnerability. However, our findings that EC S1PR1 signaling is protective in human RA are not congruent with a report showing elevated EC S1PR1 in RA synovium (72), a result that might be explained by the known increase in numbers of microvessels in hyperproliferative synovium rather than an increase in S1PR1 expression on individual ECs.

Discovery of new targets and agents that could be used in combination with standard medicines can benefit patients with RA and other autoimmune diseases. We propose development of novel antiinflammatory agents that increase vascular barrier function and attenuate tissue damage without inducing immunosuppression, such as SAR247799 (73). Because S1PR1 and other S1PRs are widely expressed, and agonists may amplify deleterious pathways, optimal therapeutic approaches would specifically maximize EC S1PR1 antiinflammatory and pro-barrier effects. Possibilities include delivery of S1PR1 agonists in nanoparticles targeting activated ECs or boosting EC S1PR1 expression in inflamed ECs by introducing S1PR1 mRNA, available technologies that can be applied to harness the protective functions of ECs.

## Methods

### Sex as a biological variable.

Our study examined male and female animals, and each group of mice was balanced according to sex and age. SIA is similar for both sexes (74).

### Mice and animal studies.

*Apom^–/–^* mice were a gift from L.B. Niesen and C. Christoffersen, Rigshospitalet, Copenhagen, Denmark. EC-specific gene deletion (ECKO) or overexpression of S1PR1 (S1PR1 GOF) was performed by crossing Cdh5-CreERT2 mice to *S1pr1*^fl/fl^ or *S1pr1^fl/stop/fl^* mice, respectively (17, 44), which were provided by Harvard School of Medicine (Timothy Hla Laboratory), Boston, Massachusetts, USA. FVN/B mice were purchased from The Jackson Laboratory. To induce Cre activity and control for the effects of tamoxifen, 8- to 12-week-old Cre-positive and Cre-negative (*S1pr1*^fl/fl^) mice were treated with tamoxifen (10 mg/mL in corn oil; 200 µL IP) once daily for 5 days. Mice with stabilized EC contacts in which the Cdh5 gene locus was targeted with a VE-cadherin–α-catenin construct, which we refer to in this manuscript as VE-cad-α-cat mice, were provided by Max Planck Institute, Munster, Germany (53).

To induce SIA, 50–100 μL K/BxN serum was injected IP into 10- to 16-week-old male and female mice on days 0 and 2 (37). Clinical arthritis scores were determined in a blinded fashion daily until day 7–8, at which time mice were sacrificed. To assess vascular leakage, Evans blue (0.5%, 150 μL) was injected by tail vein injection 1 hour prior to sacrifice. Mice used in these experiments were sacrificed on days 3, 8, or 18 for assessment of Evans blue extravasation. To quantify extravascular Evans blue extravasation, front and rear paws were isolated, minced, and treated with 1 mL of formamide, then heated to 65°C overnight. Evans blue extravasation was measured in the supernatants by absorbance at 620 nM. To bolster S1PR1 signaling in SIA-exposed mice, CYM-5442 was administered 0.25 mg/kg IP daily, with first dose administered 30 minutes prior to first K/BxN serum injection. To assess the effects of an S1PR1 antagonist, NIBR-0213 (Cayman Chemical) was administered daily IP at 30 mg/kg as described (75) on days 0–3.

To assess effects on vascular leakage in lung tissues, S1PR1 antagonist NIBR-0213 was administered (30 mg/kg, IP) (75) 2 hours prior to IV injection with Evans blue (0.5%, 150 μL). Mice were sacrificed 60 minutes thereafter. The pulmonary vasculature was perfused with 30 mL of PBS after cutting the renal artery and flushing the right ventricle with 30–60 mL of PBS. To collect BAL fluid, a 20-gauge catheter was inserted into the trachea, and 500 μL of PBS was inserted and removed a total of 3 times to collect ~1.5 mL of BAL fluid. To test whether ADAM10 mediates VE-cadherin shedding in response to S1PR1 antagonism, a modified protocol was used. Mice were injected with IV GI254023X (50 mg/kg ~ 1.25 mg in 50 μL of DMSO diluted in 100 μL of PBS/0.5% Evans blue) 30 minutes prior to administration of NIBR-0213 (10 mg/kg, IP). Mice were sacrificed for BAL isolation 30 minutes after challenge with NIBR-0213.

### Neutrophil depletion studies.

Mice were injected IP with anti-GR1 (100 μg) or anti-Ly6G (200 μg, BioXCell, BE0075 and BE0075-1, respectively) 1 day prior to testing for depletion. Because C57BL/6 mice are relatively resistant to neutrophil depletion via anti-Ly6G using this method (50), FVB/N mice were used for experiments with anti-Ly6G. To confirm neutrophil depletion, 100–200 μL of blood was collected directly into lyse-fix buffer (BD Biosciences), incubated for 10 minutes at 37°C, washed, preincubated with Fc blocking antibodies followed by anti-CD11b-PE (BioLegend, 101207), and analyzed by flow cytometry.

### Histological assessment of synovial tissues.

Skin over the ankle was cut longitudinally on both sides, and ankles were fixed in 10% formalin for 24 hours at room temperature (RT), then rinsed with PBS and decalcified in 20% EDTA, pH 7.4, for 1 week at RT prior to paraffin-embedding and sectioning (10 μM) and staining with H&E. Sections were scored by a pathologist in a blinded manner. At least 3 H&E sections were evaluated. Severity of arthritis was estimated by evaluating cartilage damage as in ref. 76 and synovial inflammation as in ref. 77. The inflammation was visually evaluated and defined as absent, 0; minimal, 1; mild, 2; moderate, 3; marked, 4; and severe, 5. Density of inflammatory cell infiltrate and spreading through periarticular soft tissues was considered. Bone resorption was graded from 0 to 5: number and size of bone trabeculae, as well as depth and integrity of cortical bone, were evaluated. Polymorphonuclear cells were counted using 3 H&E sections, on 6 original magnification, 40×, fields total.

### MicroCT evaluation of paws after SIA.

Bone volume/total volume fraction was generated using the μCT35 Scanco Medical system (software version V6.1). Contours were manually drawn over 100 stacked images for each treatment condition. Parameters were calculated for 1 mm incremental distances and averaged.

### EC studies.

HUVECs were obtained from pooled donors (Lonza) and cultured in endothelial basal media (EBM) supplemented with growth factors EGM-2 Bulletkit (Lonza) and used up to passage 8. Synovial microvascular ECs were purchased from Cell Systems and used to passage 8. Prior to experiments, ECs were serum-starved in EBM without supplements for 3 hours. Then medium was changed and treated with NIBR-0213 and/or the metalloproteinase inhibitor MM, provided by Ouathek Ouerfelli, Memorial Sloan Kettering Cancer Center, New York, New York, USA, or GI254023X (Cayman Chemical) applied at indicated concentrations. Protein lysates from confluent cultures of HUVECs were extracted using RIPA buffer (MilliporeSigma) containing phosphatase inhibitors (Roche) and protease inhibitors (Thermo Fisher Scientific) and MM, followed by centrifuging the homogenate at 13,200 RCF at 4°C for 5 minutes. Solubilized proteins generated were used for Western blot.

To knock out ADAM10 or ADAM17 from HUVECs, cells were transfected with siRNA (25 nM) 2–3 days prior to treatment with NIBR-0213. SiRNAs were diluted with the transfection reagent (DharmaFECT) in serum-free medium for 20 minutes prior to its addition to cells that were 60%–70% confluent. ADAM10 knockout was confirmed by Western blot (Supplemental Figure 10).

To isolate soluble VE-cadherin from cell supernatants or BAL fluid, samples were treated with concanavalin A beads (MilliporeSigma) to concentrate glycosylated proteins overnight at 4°C. Samples were centrifuged at 1,000*g* for 5 minutes and washed 3 times in PBS/1% Triton X-100, and beads were then boiled for 5 minutes in Laemmli sample loading buffer supplemented with mercaptoethanol. After centrifugation at 15,000*g* for 1 minute at 4°C, supernatants were collected and subjected to Western blotting.

### Western blotting.

Equal volumes of cell lysates or supernatants were loaded on 8% and 10% Tris-glycine gels for SDS-PAGE followed by transfer onto nitrocellulose or PVDF membranes. Western blots were blocked in 20 mM Tris pH 7.5, 150 mM NaCl, and 0.1% Tween 20 with 5% milk before overnight incubation with a C-terminal–specific VE-cadherin antibody (Abcam, ab33168), an N-terminal–specific VE-cadherin antibody (Santa Cruz Biotechnology, sc-52751), or to control for protein loading, an antibody against β-actin (Cell Signaling Technology, 4970). HRP-conjugated anti-mouse and anti-rabbit (Invitrogen, 31460, for anti-rabbit; 62-6520 for anti-mouse) were used at 1:5,000 in milk for 30 minutes and washed prior to treatment with ECL (SignalFire, Cell Signaling Technology).

### ECIS.

ECIS was performed as described (17). Confluent HUVECs or synovial microvascular ECs were serum-starved for 3 hours prior to treatment with NIBR-0213 (500 nM) with or without MM (1 μM), GI254023X (1 μM), or the neutralizing ADAM17 monoclonal antibody D1(A12) (150 nM; MilliporeSigma, MABT884) (55). Measurements were collected every 20 seconds for 60 minutes.

### Soluble VE-cadherin measurements.

Soluble VE-cadherin in plasma, BAL fluid, or synovial lavage fluid was measured using the mouse VE-Cadherin ELISA Kit (Abcam) according to the manufacturer’s protocol.

### FACS of ECs from joint tissue after SIA.

On day 7 of SIA, mice were sacrificed, and paws were excised, minced, and suspended in 5 mL of PBS containing Liberase (Roche, 5 μg/mL) and DNase (MilliporeSigma, 200 μg/mL) at 37°C with shaking for 60 minutes. Postdigestion, 5 mL of DMEM containing 20% FBS was added to each sample. Cell suspension was filtered through a 40 μm cell strainer (Falcon) and centrifuged at 600*g* for 5 minutes at 4°C. Cells were resuspended in wash buffer containing DMEM supplemented with 20% FBS+EDTA (1 μM) and blocked with anti-mouse CD16/CD32 (1:250, BD Biosciences, 553141) for 10 minutes at 4°C. Cells were then incubated for 1 hour at 4°C with anti–CD31-APC (1:100, BioLegend, 102409) and anti–CD45-PE (1:100, BioLegend, 147711), washed, and labeled with DAPI to identify dead cells. The CD31^+^CD45^–^DAPI^–^ (>95% purity) population was sorted using FACS (BD Vantage Cell). Cells were sorted directly into RLT lysis buffer (QIAGEN) and stored at –80°C.

### EC RNA isolation for RNA-Seq and data analysis.

Total RNA was extracted using RNeasy Micro Kit (QIAGEN). Library preparation and sequencing were performed at the Epigenetics Core Facility at Weill Cornell Medicine using the SMART-Seq v4 Ultra Low Input RNA Kit (Takara). The sequencing libraries were sequenced with paired-end 50 bp on NovaSeq 6000 sequencer (Illumina). The raw sequencing reads in BCL format were processed through bcl2fastq 2.20 (Illumina) for FASTQ conversion and demultiplexing. After trimming the adaptors with cutadapt (version 1.18), RNA reads were aligned and mapped to the GRCh38 human reference genome by STAR (Version 2.5.2) (78), and transcriptome reconstruction was performed by Cufflinks (Version 2.1.1). The abundance of transcripts was measured with Cufflinks in fragments per kilobase of exon per million mapped reads (FPKM) (79, 80). Gene expression profiles were constructed for differential expression (DE) analysis with the DESeq2 package (81). Differentially expressed genes (DEGs) were defined as those with average FPKM > 1 in either the WT or S1PR1-ECKO group and *P* < 0.004. DEGs up- and downregulated in the S1PR1-ECKO group were independently input to Database for Annotation, Visualization and Integrated Discovery for Kyoto Encyclopedia of Genes and Genomes pathway analysis. FPKM values for transcripts belonging to selected pathways with *P* < 0.0004 were input to heatmap.2 software for visualization of expression *z* scores. Volcano plot of genes from DE analysis was rendered in GraphPad Prism software. To ensure that leukocyte transcripts did not contaminate the EC RNA-Seq data, we confirmed that the FPKM for PTPRC (encoding CD45) was less than 1 in 7/8 samples and less than 1.5 in all the samples analyzed.

### Quantitative sphingolipid determination.

Sera samples were obtained from the Hospital for Special Surgery. We studied sera from 20 patients with RA who fulfilled the American College of Rheumatology 2010 Rheumatoid Arthritis classification criteria and 20 age- and sex-matched individuals with OA.

Sphingolipids were quantified by high-pressure liquid chromatography electrospray ionization tandem mass spectrometry (HPLC-MS/MS) using minor modification of a described method validated for S1P and sphinganine-1 P (82). Serum samples (25 μL) were extracted in dichloromethane methanol (1:1) with addition of 25 pmol internal standard (N-lauroyl-D-erythro-sphingosylphosphorylcholine) and then centrifuged (4,000*g*, 10 minutes, 4°C) to precipitate cell debris. Samples were injected into an Agilent 1200 HPLC system equipped with Agilent Poroshell 120 EC C18 column linked to an Agilent 6430 triple-quadrupole mass spectrometer.

MassHunter optimizer and pure synthetic standards (Avanti Polar Lipids) were used to determine optimum fragmentation voltage, precursor/product ions, and *m/z* values. Peak calls and abundance calculations were obtained with MassHunter Workstation Software (Agilent). Final concentrations are calculated from a standard curve for each sphingolipid run in parallel.

### Analysis of human single-cell RNA-Seq data.

For single-cell RNA-Seq comparisons of endothelial S1PR1 in synovial tissues, we compared transcripts previously collected from 3 data sets: (i) RA individuals from the AMP RA/SLE network (57); (ii) RA individuals from the Roche network, described in Korsunsky et al. (58); and (iii) healthy controls from Faust et al. (59).

The Seurat package (v4.3.0) implemented in R was used for analysis of 3 single-cell RNA-Seq data sets generated from human synovial tissue. The gene-cell count matrices and available metadata were loaded into R. If available, existing metadata were used to subset each data set to contain only synovial ECs. The standard Seurat analysis pipeline was followed through principal component analysis (PCA). Following PCA, the Harmony package (v0.1.1) implemented in R was used to integrate the 3 data sets together across their respective sample origins. After data integration, Harmony-corrected principal components were used for downstream analysis. Cluster assignment was carried out using Seurat’s “FindClusters” function at a resolution of 0.5. Cluster identities were then annotated based using the top 10 marker genes for each cluster.

After annotating the integrated data set with its respective EC type identities, capillaries were then subsetted for downstream visualization of S1PR1 expression. Seurat’s “FetchData” function was used to extract expression data for S1PR1 and relevant metadata fields from the capillary subset. Visualization was performed using the ggpubr package (v0.6.0) implemented in R. *P* values were calculated with ggpubr’s “stat_compare_means” function based on a series of pairwise Wilcoxon rank-sum tests across RA statuses.

### Statistics.

The data were analyzed and graphs were generated using GraphPad Prism 9. Two-tailed Student’s *t* tests, 1-way ANOVA with Tukey’s post hoc tests, and Wilcoxon rank-sum tests were performed as indicated. A *P* value less than 0.05 was considered significant.

### Study approval.

Animal experiments were performed under the guidelines set by the Institutional Animal Care and Use Committee at Weill Cornell Medicine. For human samples, written informed consent from the donors was obtained. Sera were used in full agreement with the approval of the Institutional Review Board (IRB) at the Hospital for Special Surgery. AMP, Roche, and healthy data sets were used in full agreement with the corresponding institutional IRBs.

### Data availability.

The authors confirm that the data associated with the manuscript and supplemental material are provided in a single Supporting Data Values XLS file in the supplemental material. Bulk RNA-Seq data from Supplemental Figure 3 have been deposited at Zenodo (DOI: 10.5281/zenodo.11075064; https://zenodo.org/doi/10.5281/zenodo.11075064).

## Author contributions

NB conceived the study. NB, JES, and CB designed research studies; NB, RM, LA, AK, KA, and MT conducted experiments and acquired data; NB, EE, JES, TW, TH, HJF, MB, MT, and KW analyzed data; BM and SG contributed reagents; MM designed the graphical abstract; TP performed the histological scoring; NB and JES wrote the manuscript; and TH, DV, HJF, MB, KW, and CB edited the manuscript.

## Supplementary Material

Supplemental data

Unedited blot and gel images

Supporting data values

## Version 1 06/10/2024

Electronic publication
