# Supplementary material for: Endothelial cell sphingosine 1-phosphate receptor 1 restrains VE-cadherin cleavage and attenuates experimental inflammatory arthritis
Source: JCI Insight. 2024 Jun 10;9(11):e171467. doi: 10.1172/jci.insight.171467 (PMC11382883; doi:10.1172/jci.insight.171467)
Supplement: Supplemental data [file jciinsight-9-171467-s071.pdf]

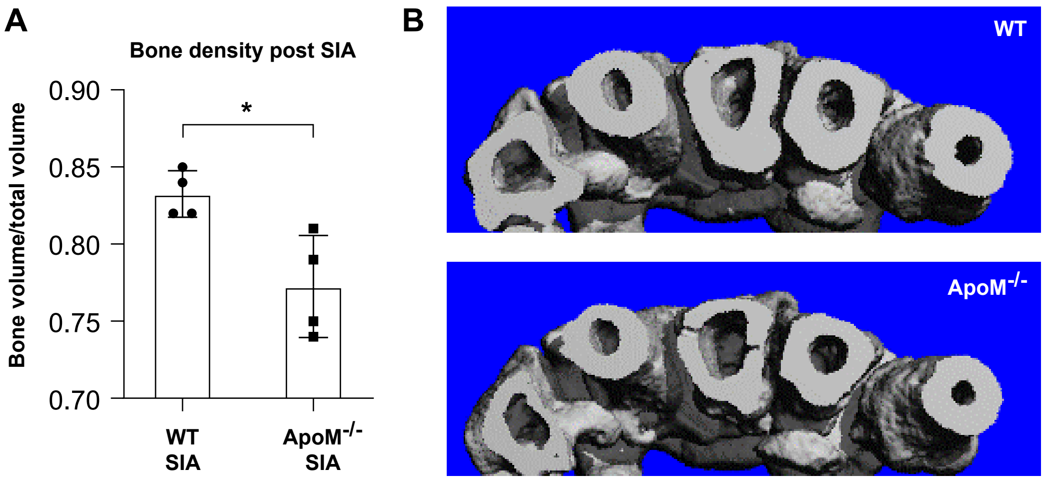

Supplemental data 1. ApoM-/- Mice demonstrate decreased bone volumes by micro CT after SIA. Each point corresponds to a paw from an individual mouse. Significance was calculated using the unpaired Student's t test. Values are the mean ± SEM. n=4; *p<0.05

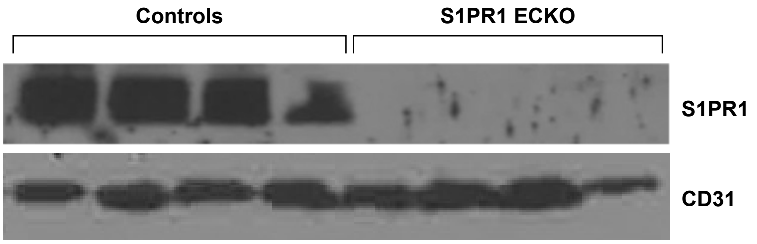

Supplemental data 2. Confirmation of S1PR1 KO after tamoxifen treatment: lung lysates from S1PR1 ECKO mice and littermate controls were probed with anti-S1PR1 antibody (above) and anti-CD31 as loading control (below).

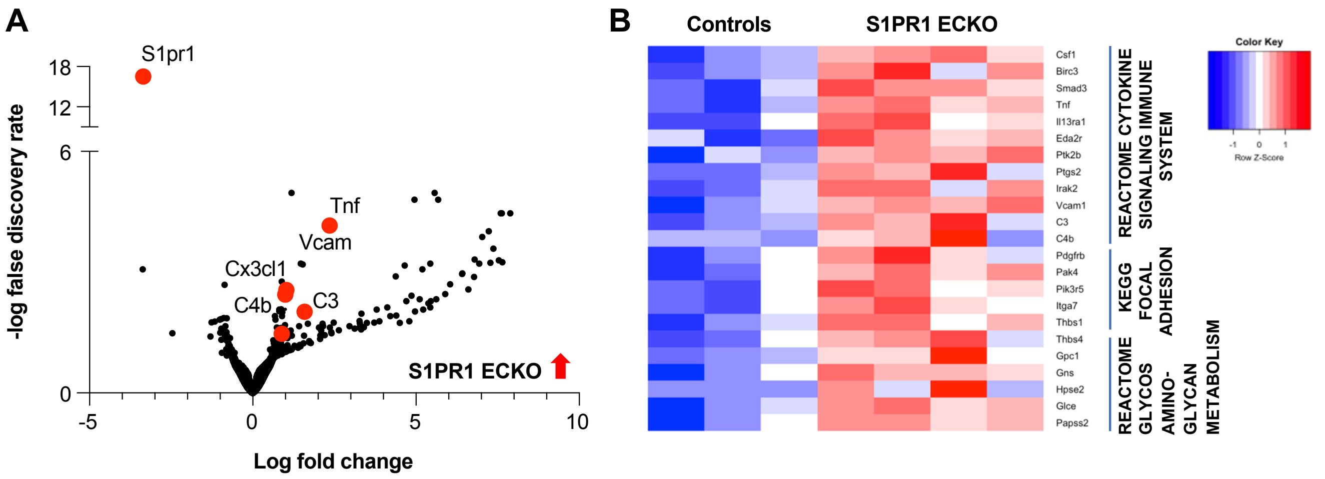

Supplemental Data 3. Deletion of endothelial S1PR1 increases endothelial transcripts of pro-inflammatory mediators known to contribute to SIA. Flow sorted CD45-/CD31+ endothelial cells from inflamed synovial tissues from S1PR1 ECKO and control mice (SIA day 8) were analyzed by RNAseq. (A) Volcano plot of Differential Gene Expression (DEG) in EC S1PR1 ECKO mice vs controls highlighting inflammatory genes involved in SIA. Red circles indicate upregulated or downregulated genes. Note S1PR1 deletion. (B) Heat map representation of DEG and corresponding KEGG analysis.

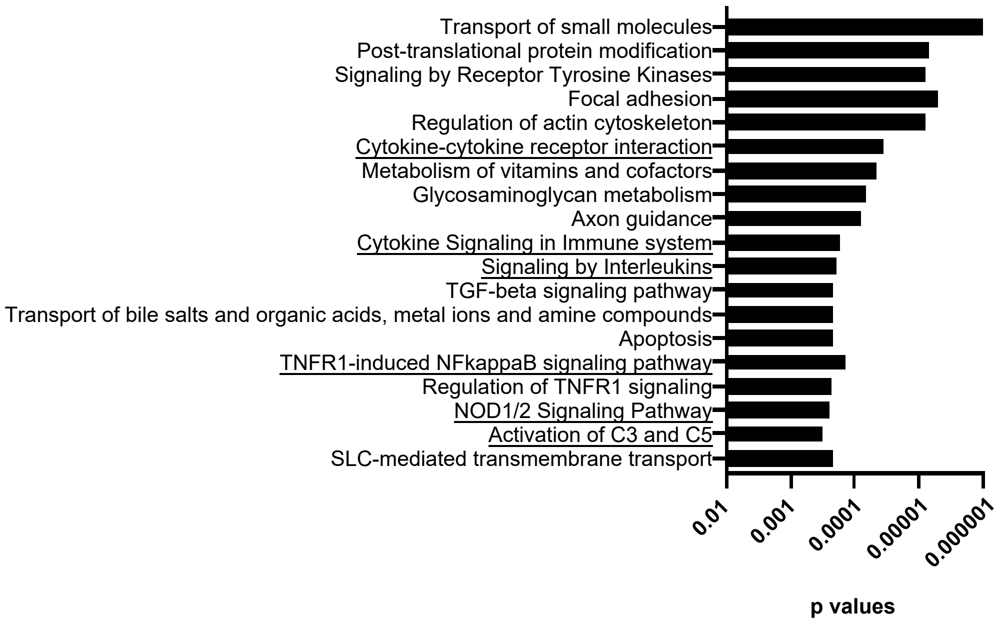

Supplemental data 4. Kegg pathway analysis of upregulated genes in S1PR1 ECKO synovial ECs at day 7 of SIA. Inflammatory pathways are underlined.

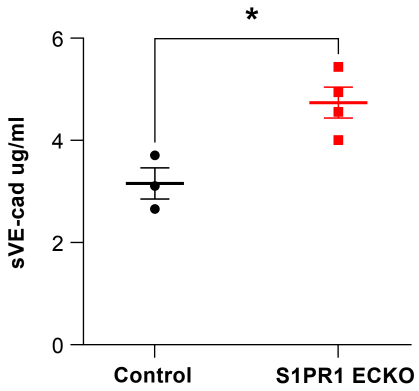

Supplemental data 5. Plasma levels of soluble VE-cadherin in S1PR1 ECKO mice and controls at baseline. Significance was calculated using the unpaired Student’s t test. Values are the mean ± SEM; n= 3-4 mice/group from 2 independent experiments. *$p<0.05$

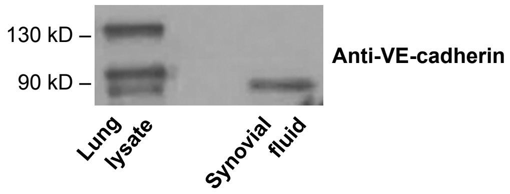

Supplementary data 6. Synovial fluid from SIA treated mice reveals only the 90 kD form of VE-cadherin. Perfused lung lysates show full length and cleaved forms.

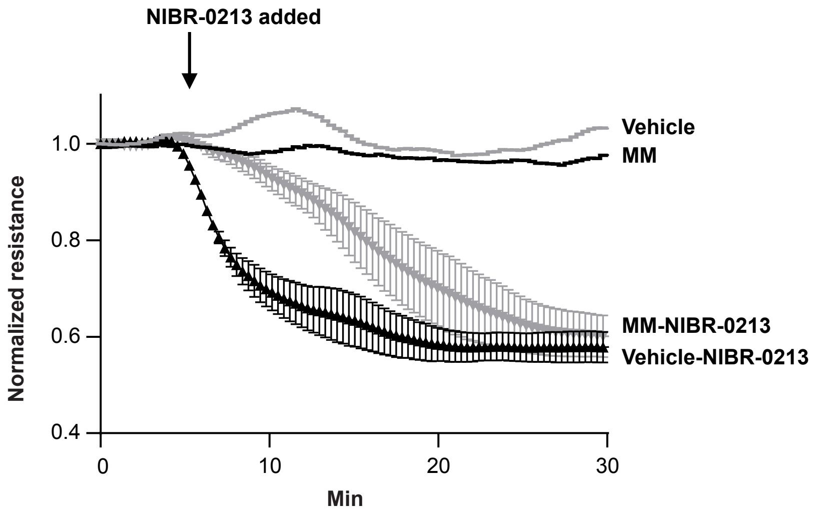

Supplemental data 7: Inhibition of metalloproteinase limits barrier dysfunction induced by S1PR1 blockade in primary synovial endothelial cells (SECs). SECs were treated with the metalloproteinase inhibitor Marimastat (MM, 1uM) or DMSO for 30-60 min prior to treatment with NIBR-0213. Resistance across confluent ECs was measured by ECIS. Each condition was run in triplicate.

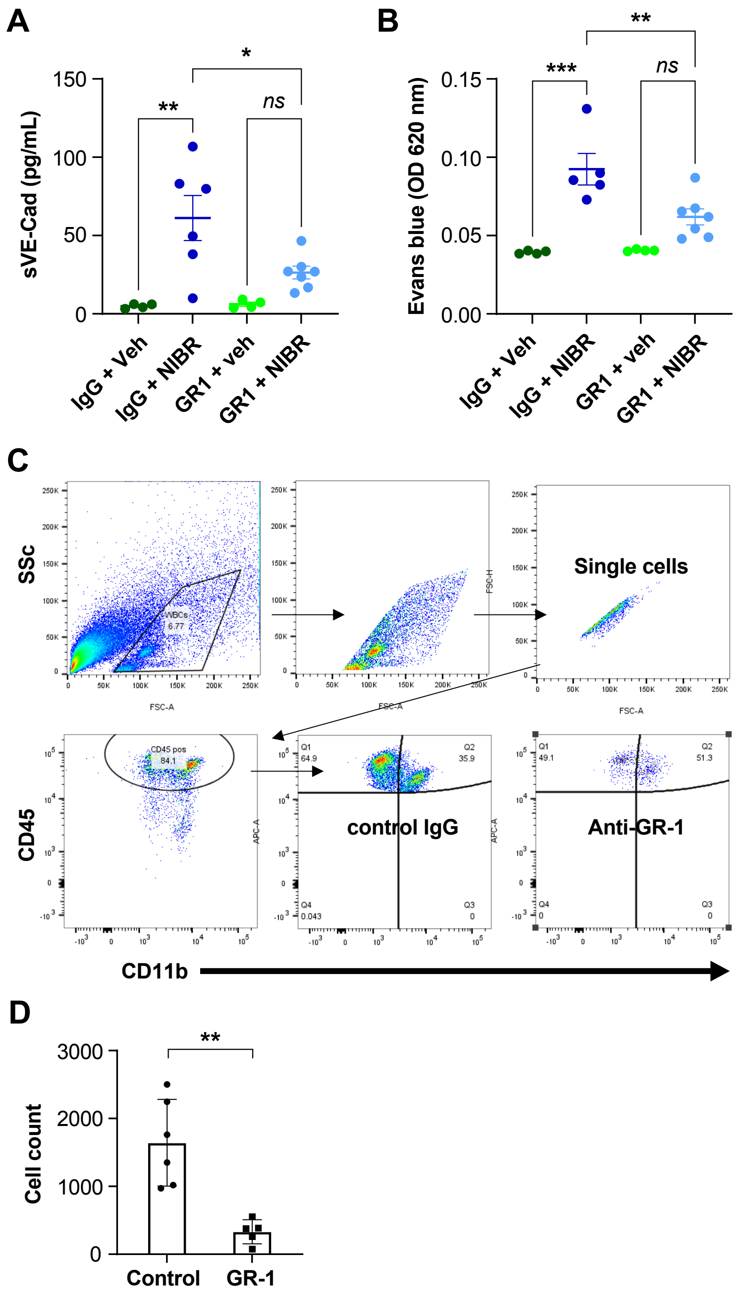

Supplemental data 8. S1PR1 blockade induces VE-cadherin shedding and vascular leakage in lung in mice treated with anti-GR1 but less so than after anti-Ly6G. (A) Soluble VE-cadherin and (B) Evans blue in BAL fluids of mice treated with isotype control or anti-GR1 one day prior to treatment with NIBR-0213. (C) Flow cytometry confirmation that myeloid cells were depleted after treatment with anti-GR1. (D) Quantitation of CD11b+ cells after GR-1 treatment vs. isotype control. Statistical test was 1-way ANOVA and Tukey's post hoc test (A and B) and unpaired student T test (D); * $p<0.05$; ** $p<0.01$; *** $p<0.001$; ns, not significant, or as indicated.

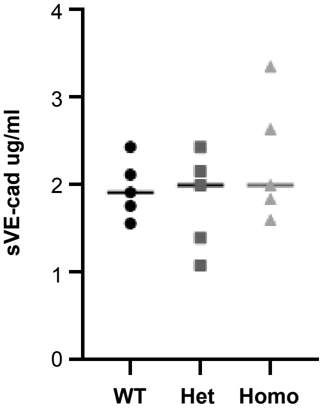

Supplemental data 9. Homozygous VE-cadherin-alpha-cat mice have similar circulating levels of soluble VE-cadherin compared to heterozygous and WT controls.

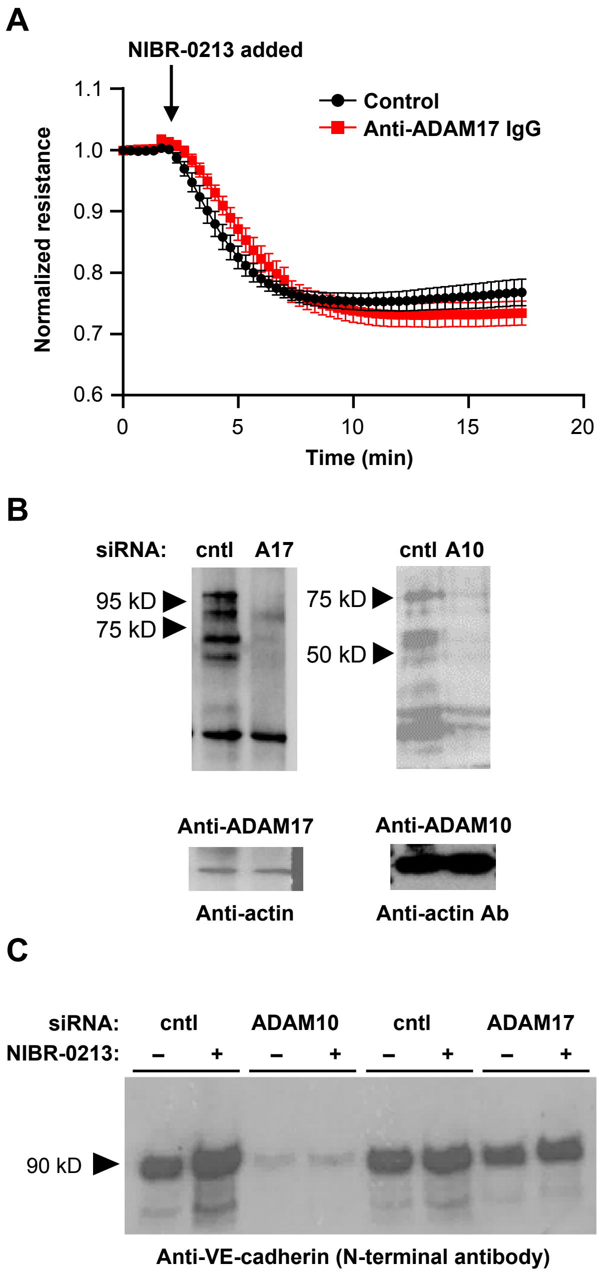

Supplemental data 10. ADAM17 is not required for NIBR-0213 induced EC barrier dysfunction or shedding of VE-cadherin. (A) HUVEC were treated with neutralizing antibody to ADAM17(D1A12) or PBS 30 min prior to addition of NIBR-0213 and resistance across confluent ECs was measured in ECIS; n=3, p=ns. (B) HUVEC were treated with siRNA to ADAM17 or ADAM10 for 24-48 hrs and lysates were analyzed by western blot to confirm knock down. Top panels: western blot of HUVEC lysates probed with ADAM17 and ADAM 10 antibodies, respectively. Bottom panels: western blots were probed with anti-actin antibodies as a loading control. (C) HUVEC treated with control, ADAM10, or ADAM17 siRNA were treated with NIBR-0213 (10 uM) for 1hr. Supernatants were collected, treated with concanavalin A beads to concentrate soluble proteins and subjected to western blotting and probed with anti-VE-cadherin antibodies (N-terminal specific).

Condition	RA (n = 20)	OA (n = 20)
**Age (mean + SD)**	63.8 + 11.9	64.5 + 5.9
**Gender (% female)**	99	100
**Disease duration (yr)**	15.3 + 11.2	unknown
**RF+**	50%	NA
**CCP+**	70%	NA
**DAS (mean + SD)**	4.6 + 0.86	NA
**Methotrexate**	40%	NA
**Any biologic**	50%	NA

Supplemental data11: Clinical data from RA and OA subjects included for serum measurements of S1P and Sa1P.
