# Supplementary material for: Endothelial cell sphingosine 1-phosphate receptor 1 restrains VE-cadherin cleavage and attenuates experimental inflammatory arthritis
Source: JCI Insight. 2024 Jun 10;9(11):e171467. doi: 10.1172/jci.insight.171467 (PMC11382883; doi:10.1172/jci.insight.171467)
Supplement: Unedited blot and gel images [file jciinsight-9-171467-s072.pdf]

Uncropped Western Blots

Burg et al, Endothelial cell Sphingosine 1-Phosphate Receptor 1 restrains VE-cadherin cleavage and attenuates experimental inflammatory arthritis

Figure 3G (boxed area is in figure)

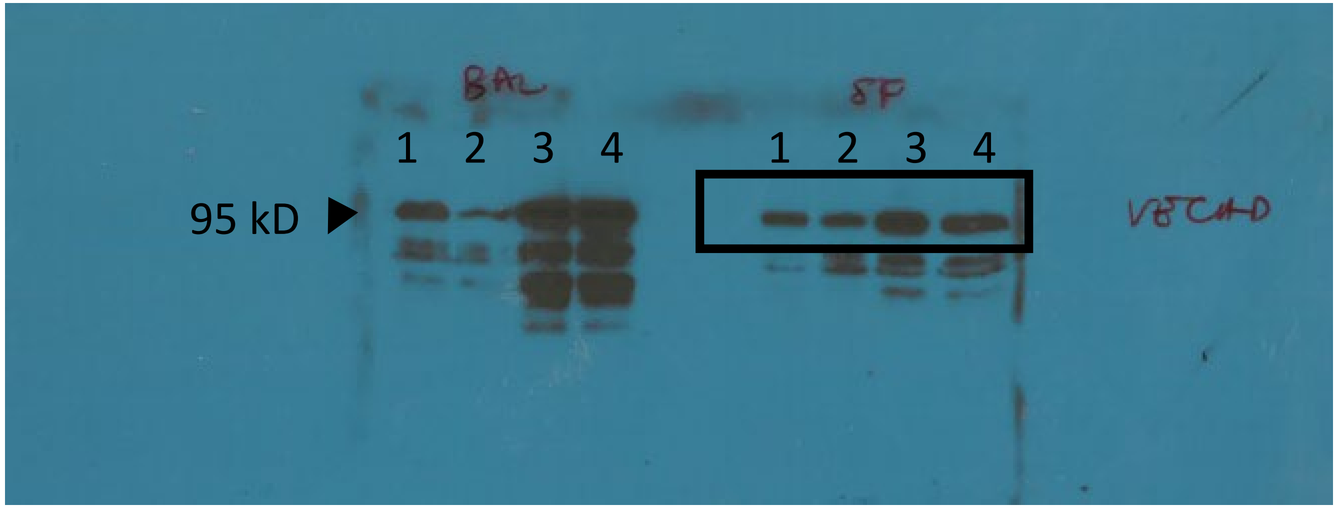

Probed with Anti-VE-cadherin BV9 (N-terminus specific)

1 BAL control mouse 1
2 BAL control mouse 2
3 BAL S1PR1 ECKO mouse 1
4 BAL S1PR1 ECKO mouse 2

1 synovial lavage control mouse 1
2 synovial lavage control mouse 2
3 synovial lavage S1PR1 ECKO mouse 1
4 synovial lavage S1PR1 ECKO mouse 2

Figure 4B (entire blot in figure)

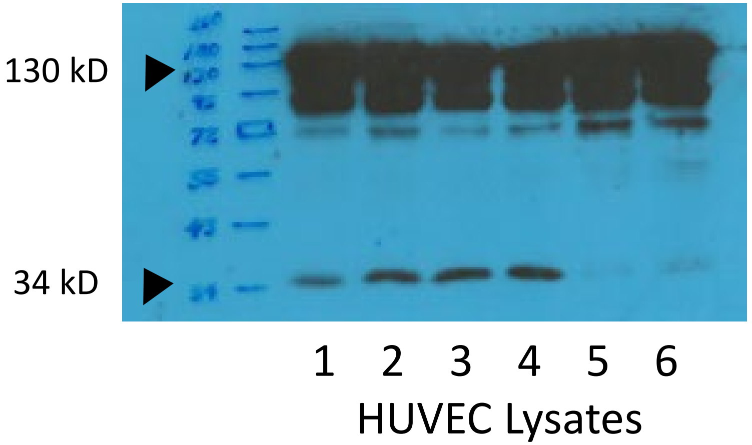

Probed with Anti-VE-cadherin AB (C-terminus specific)

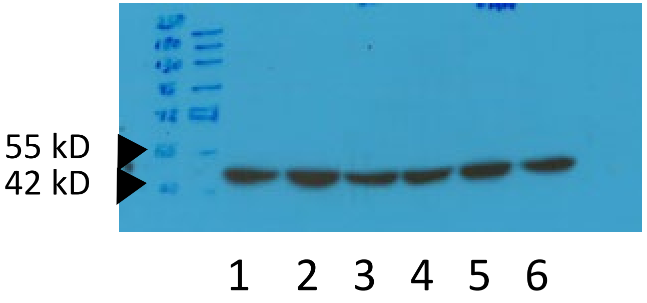

Probed with Anti-actin AB

1 control
2 NIBR (10 uM) 30 min
3 NIBR (10 uM) 60 min
4 NIBR (10 uM) 180 min
5 NIBR (10 uM) 180 min + Marimastat
6 180 min control + Marimastat

Figure 4C (boxed area is in figure)

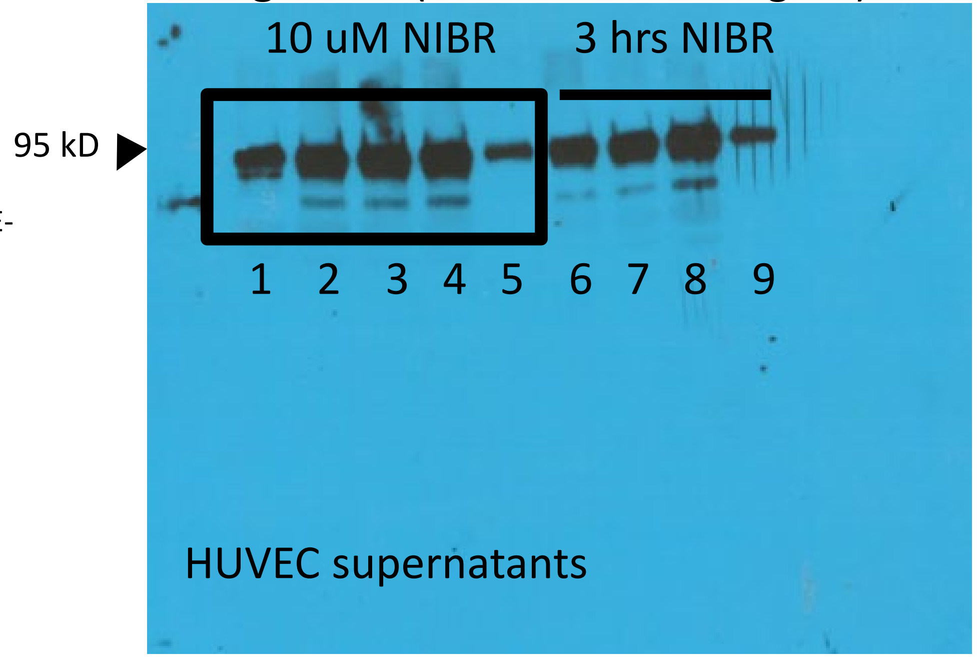

Probed with Anti-VE-cadherin AB (N-terminus specific)

1 control
2 NIBR (10 uM) 30 min
3 NIBR (10 uM) 60 min
4 NIBR (10 uM) 180 min
5 NIBR (10 uM) 180 min + Marimastat

6 NIBR (3hr) 1uM
7 NIBR (3hr) 5 uM
8 NIBR (3hr) 10 uM
9 NIBR (3hr) 10 uM + Marimastat

Figure 5B (boxed area in figure)

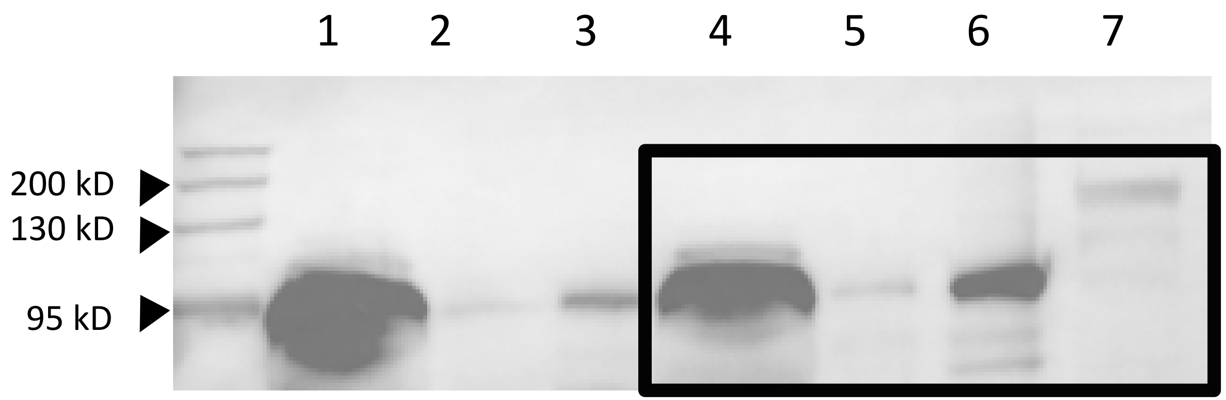

1 Plasma mouse 1
2 BAL fluid baseline mouse1
3 BAL fluid after NIBR-0213 mouse1
4 Plasma mouse 2
5 BAL fluid baseline mouse 2
6 BAL fluid after NIBR-0213 mouse 2
7 HUVEC lysate (to show full length VE-cadherin)

Supplemental data 2 (boxed areas in figure)

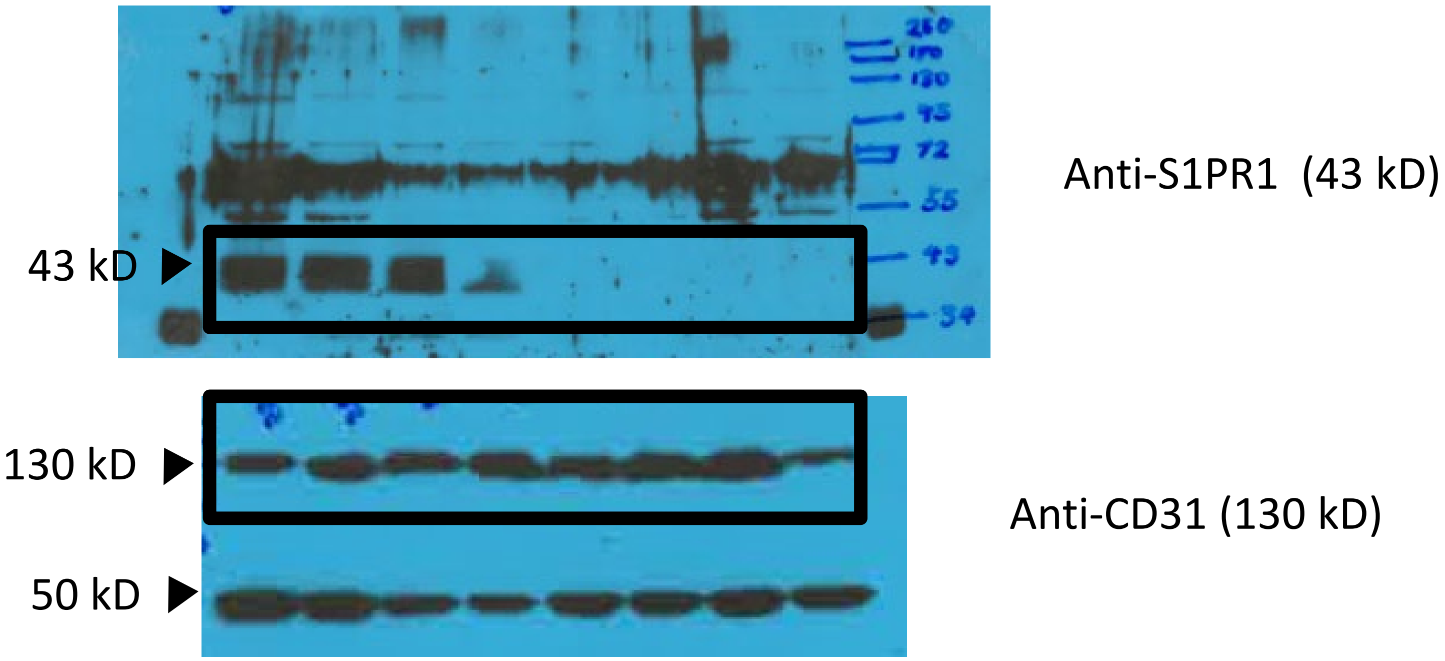

1 S1PR1 f/f 1
2 S1PR1 f/f 2
3 S1PR1 f/f 3
4 S1PR1 f/f 4
5 S1PR1 ECKO 1
6 S1PR1 ECKO 2
7 S1PR1 ECKO 3
8 S1PR1 ECKO 4

Supplemental data 6 (boxed area in figure)

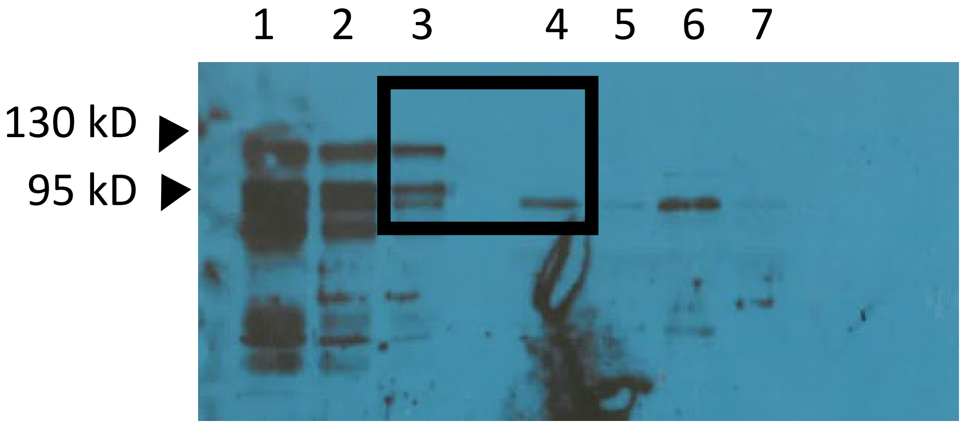

Anti-VE-cadherin (BV9) N-terminus specific

1 Lung lysate 1
2 lung lysate 2
3 lung lysate 3
4 synovial fluid 1
5 synovial fluid 2
6 synovial fluid 3
7 synovial fluid 4

Supplemental data 10 B (boxed areas in figure)

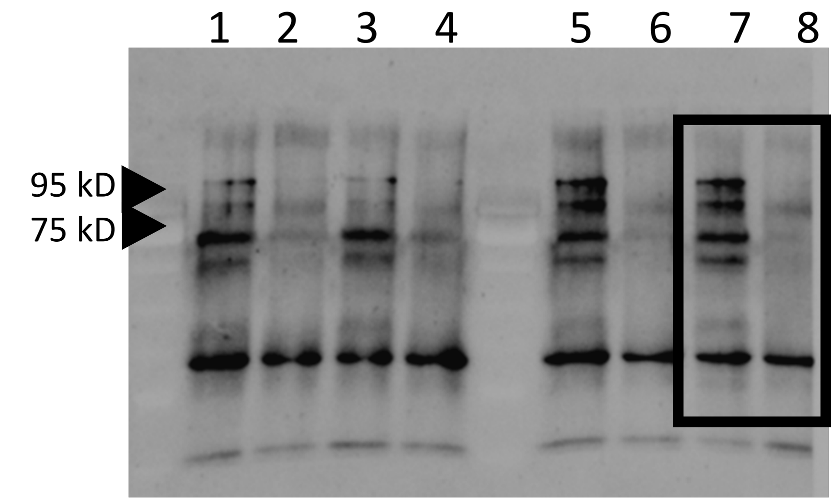

Anti-ADAM17

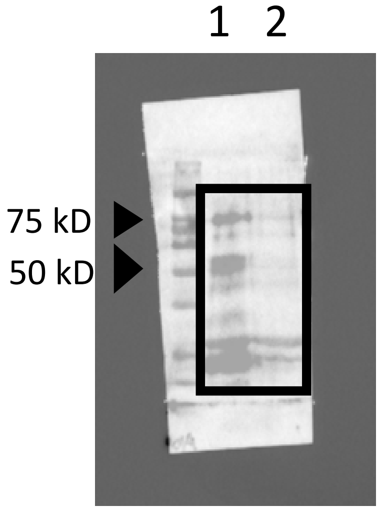

Anti-ADAM10

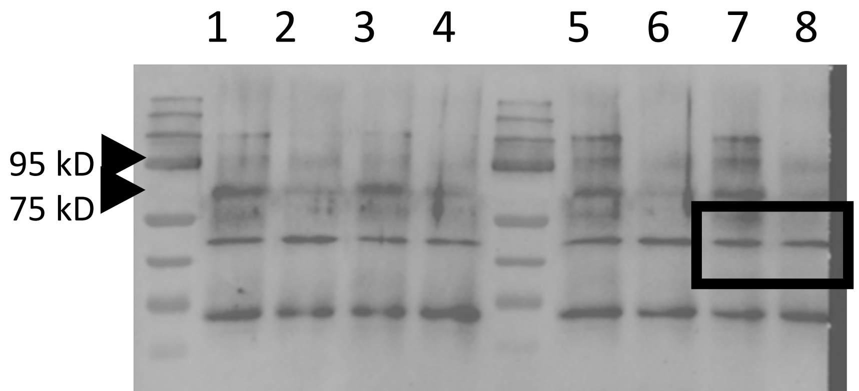

Anti- beta actin

1,3,5,7. control siRNA
2,4,6,8. ADAM17 siRNA

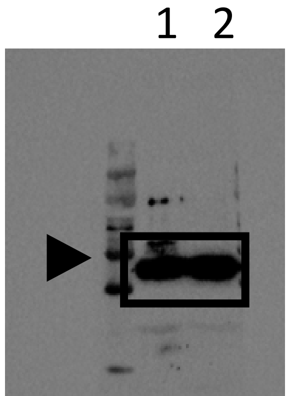

Anti- beta actin

1. control siRNA
2. ADAM 10 siRNA

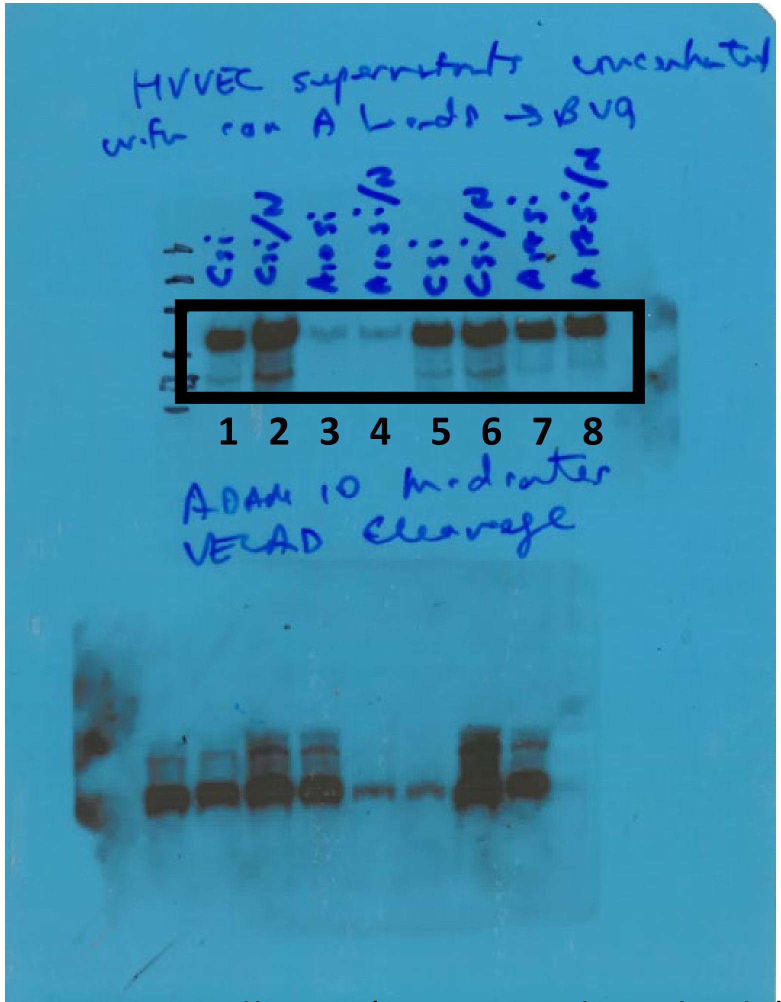

Anti-VE-cadherin (N-terminal antibody)

1 cntl siRNA
2 cntl siRNA+NIBR
3 ADAM10 siRNA
4 ADAM10 siRNA+NIBR
5 cntl siRNA
6 cntl siRNA+NIBR
7 ADAM17 siRNA
8 ADAM17 siRNA+NIBR

Bottom- longer exposure, flipped
